# Ameliorative effects of a *Lactobacillus paracasei* and *Puerariae Radix* extract complex on hydrogen peroxide-induced oxidative damage in zebrafish

**DOI:** 10.3389/fphar.2026.1787487

**Published:** 2026-06-17

**Authors:** Suyu Chen, Ting Tang, Yang Junhong, Liu Bosi, Dianpeng Wang, Dafeng Lin, Xiangli Yang, Peimao Li, Wen Zhang, Naixing Zhang

**Affiliations:** 1 Medical Laboratory, Shenzhen Prevention and Treatment Center for Occupational Diseases, Shenzhen, China; 2 School of Public Health, Southern Medical University, Guangzhou, China; 3 School of Public Health, Huazhong University of Science and Technology, Wuhan, China

**Keywords:** anti-inflammatory effect, *Lactobacillus* paracasei, oxidative damage, Puerariae, Zebrafish

## Abstract

**Objective:**

This study aimed to investigate the protective effects of a novel composite comprising *Lactobacillus* paracasei and *Puerariae Radix* extract (Lac_PRE) against hydrogen peroxide (H_2_O_2_)-induced multi-organ oxidative damage in a zebrafish model.

**Methods:**

An acute toxicity test was conducted to determine the optimal subacute H_2_O_2_ exposure concentration in wild-type AB strain zebrafish. Subsequently, zebrafish were divided into four groups: control, H_2_O_2_ exposure (2.0 mmol/L), H_2_O_2_ exposure with Lac_PRE treatment, and Lac_PRE control. After 14 days, we assessed hepatic and cardiac function markers, inflammatory gene expression (cyclooxygenase, cox; tumor necrosis factor-alpha, tnf-α; interleukin-6, il-6), oxidative stress indicators (malondialdehyde, MDA), and the intestinal Firmicutes/Bacteroidetes (F/B) ratio.

**Results:**

H_2_O_2_ exposure induced significant oxidative and inflammatory damage in zebrafish, Kruskal-Wallis tests revealed significant overall differences among groups for hepatic ALT, hepatic MDA, cardiac cTnI, cardiac MDA, cardiac COX, cardiac TNF-α, cardiac IL-6, and the intestinal F/B ratio (all p < 0.05). Two a priori planned comparisons were tested using Dunn’s post hoc test with Bonferroni ×2 correction. H_2_O_2_ exposure significantly elevated cardiac cTnI (adjusted p = 0.002), cardiac MDA (adjusted p = 0.015), cardiac COX (adjusted p = 0.029), TNF-α (adjusted p = 0.023), IL-6 (adjusted p = 0.005), and significantly disrupted the F/B ratio (adjusted p = 0.012) compared to control. The Lac_PRE composite significantly reduced hepatic ALT (adjusted p < 0.001) and cardiac COX expression (adjusted p = 0.023). For the remaining indicators, the H_2_O_2__Lac_PRE group showed consistent numerical improvements, but these differences did not reach statistical significance after Bonferroni correction.

**Conclusion:**

The L. paracasei and *Puerariae Radix* extract composite effectively alleviates H_2_O_2_-induced oxidative inflammatory damage in zebrafish by enhancing antioxidant defenses, suppressing inflammation, and modulating gut microbiota. These preliminary findings suggest that this probiotic-botanical combination may exert synergistic protective effects against oxidative damage. However, given the methodological limitations including small effective sample sizes, these results should be interpreted as exploratory and hypothesis-generating. Further validation in studies with larger sample sizes and individual-level analyses is warranted before translational applications can be considered.

## 1 Introduction

Oxidative stress, as a central pathological link connecting environmental exposure to the development and progression of chronic diseases, is fundamentally characterized by a dynamic imbalance between the generation and scavenging of intracellular reactive oxygen species (ROS) (Jomova et al., 2023). Excessive accumulation of ROS can attack membrane phospholipids, triggering lipid peroxidation, damage DNA double-stranded structures, induce protein carbonylation, and ultimately lead to cellular apoptosis or pyroptosis. This process has been confirmed as a common driving mechanism for aging, type 2 diabetes, atherosclerosis, and various cancers (Wang et al., 2023; Dumitru et al., 2025; Lee et al., 2025). In modern society, factors such as environmental pollution (e.g., PM2.5, heavy metal exposure), high-sugar and high-fat diets, chronic psychological stress, and electromagnetic radiation continuously exacerbate the population’s oxidative stress burden, leading to a global rising trend in the incidence of oxidative damage-related diseases. Hydrogen peroxide (H_2_O_2_), as a key member of ROS, is not only a major endogenous form of ROS produced by metabolism but also a widespread environmental oxidant (Hossain et al., 2015; Goudarzi et al., 2023; Zhang et al., 2024). Given that H_2_O_2_-induced cellular damage mimics the shared pathological basis of many chronic diseases, exploring natural intervention strategies that can effectively counteract H_2_O_2_-induced oxidative damage holds significant public health importance for the prevention of related disorders.

The heart and liver, being metabolically active organs with high energy demands, are particularly susceptible to oxidative stress damage due to their high mitochondrial density and substantial ROS production (Arumugam et al., 2023; Paillard et al., 2025). During myocardial injury, ROS-mediated increases in membrane permeability lead to the release of creatine kinase-MB (CK-MB) into the bloodstream (Jiang et al., 2021). Concurrently, the activity of superoxide dismutase (SOD) – a crucial enzyme for scavenging superoxide anions – becomes depleted (Sharma et al., 2020), and malondialdehyde (MDA), an end-product of lipid peroxidation, accumulates significantly [(Lankin et al., 2022). These three factors collectively constitute characteristic biochemical markers of myocardial oxidative damage. In contrast, hepatocellular oxidative damage is typically characterized by a marked elevation of alanine aminotransferase (ALT) (Ramachandran and Jaeschke, 2021), a mechanism closely associated with ROS-induced mitochondrial dysfunction (e.g., inhibition of Complex I/III activity) and glutathione (GSH) depletion in hepatocytes (Sreekumar et al., 2020). For instance, in alcoholic liver injury or ischemia-reperfusion injury, the release of ALT is significantly positively correlated with the degree of hepatocellular oxidative damage] (Gu et al., 2025).

Natural botanical medicines have demonstrated unique value in the field of antioxidant research due to their multi-target and low-toxicity advantages. Kudzu root (Pueraria lobata), a medicinal and edible plant, has garnered research interest. The core active metabolite of its root extract (PRE), puerarin (chemical structure: 8-β-D-glucopyranosyl-4′,7-dihydroxyisoflavone), has become a recent research focus owing to its combined free radical scavenging capacity and phytoestrogenic activity (Zhou et al., 2019; Wang et al., 2022). Studies have confirmed that puerarin can activate the nuclear factor erythroid 2-related factor 2 (Nrf2)/antioxidant response element (ARE) pathway, upregulate the expression of antioxidant enzymes such as SOD and glutathione peroxidase (GSH-Px), and simultaneously inhibit the release of pro-inflammatory cytokines (e.g., IL-6, TNF-α) mediated by nuclear factor-kappa B (NF-κB) (Zhang et al., 2023; Zou et al., 2023). It has shown significant protective effects in models of myocardial ischemia-reperfusion injury and diabetic liver injury (Ding et al., 2023). Puerarin can also alleviate ovalbumin (OVA)-induced pulmonary inflammation and delayed-type hypersensitivity by modulating Th1/Th2 immune balance, suggesting its potential role in regulating oxidative stress-related inflammation (Zhang et al., 2022). Furthermore, puerarin exhibits notable efficacy in improving microcirculation and protecting mitochondrial structural integrity, effectively mitigating mitochondrial-dependent apoptosis. Despite its significant antioxidant activity, the clinical translation potential of puerarin is limited by issues such as low oral bioavailability and short *in vivo* retention time for single-metabolite administration.

However, the clinical application of single active metabolites is often constrained by issues like low bioavailability and limited target sites. Probiotics, as important regulators of gut microbiota, offer a novel strategy to overcome the bioavailability bottleneck of phytomedicines (Wu et al., 2023). *Lactobacillus paracasei* can not only directly scavenge ROS by secreting metabolites such as exopolysaccharides and short-chain fatty acids (SCFAs) but also indirectly reduce systemic oxidative-inflammatory responses by strengthening intestinal epithelial tight junctions (e.g., upregulating occludin, ZO-1 expression), thereby reducing endotoxin influx into the bloodstream (Han et al., 2025). Previous research has shown that lipoteichoic acid secreted by *Lactobacillus* plantarum ZJ316 can directly bind to and effectively inhibit the activation of pro-inflammatory signaling pathways (e.g., NF-κB pathway) in intestinal cells, reducing the release of cytokines like IL-6 and TNF-α, thus achieving significant anti-inflammatory effects. This direct interaction can also upregulate the expression of tight junction proteins (e.g., occludin, ZO-1) in intestinal cells, enhancing the integrity of intercellular connections and reducing intestinal permeability, ultimately protecting intestinal barrier function (Gu et al., 2023). More importantly, the β-glucosidase produced by *L. paracasei* A221 can convert kaempferol-3-O-sophoroside from kale into free kaempferol, significantly enhancing its transmembrane absorption efficiency (permeability increased by 2.3-fold in a Caco-2 model). Free kaempferol activates the AKT-GSK3β-Nrf2 pathway, boosting antioxidant enzyme activity and reducing lipid peroxidation (Shimojo et al., 2018). This synergistic “probiotic-phytochemical” model provides a theoretical basis for developing novel antioxidant formulations.

The zebrafish (*Danio rerio*), recognized as a standardized model organism by the FDA, is an ideal model for oxidative damage mechanism research and drug screening due to its high genetic homology with humans (87%), transparent embryos facilitating real-time observation, short reproductive cycle (2–3 months), and low maintenance cost (Fang and Miller, 2012). In H_2_O_2_-induced oxidative damage models in zebrafish, controlled and reversible cellular damage (e.g., lipid peroxidation, protein oxidation) can be established by modulating H_2_O_2_ concentration, simulating pathological processes of human oxidative stress-related diseases.

Therefore, we hypothesized that a composite of Lac_PRE would exert synergistic protective effects against H_2_O_2_-induced oxidative damage through a dual mechanism: directly enhancing the endogenous antioxidant system and modulating the gut microbiota.

To test this hypothesis, this study utilized a zebrafish model of H_2_O_2_-induced oxidative stress with the following specific objectives: (1) to evaluate the protective effects of Lac_PRE on H_2_O_2_-induced cardiac and hepatic dysfunction by measuring biochemical markers (cTnI, CK-MB, ALT) and oxidative stress indicators (MDA); (2) to assess the anti-inflammatory effects of Lac_PRE by quantifying the expression of inflammatory genes (cox, tnf-α, il-6) in cardiac and hepatic tissues; and (3) to investigate the modulatory effects of Lac_PRE on gut microbiota homeostasis by analyzing the Firmicutes/Bacteroidetes (F/B) ratio.

## 2 Materials and methods

### 2.1 Reagents and instruments

#### 2.1.1 Main reagents

Hydrogen peroxide (H_2_O_2_): Analytical grade, 3% v/v, Guangdong Hengjian Pharmaceutical Co., Ltd. Pueraria lobata (Kudzu root) (Willd) Ohwi [Fabaceae; Puerariae lobatae radix] Purchased from China National Medicines Guoda Pharmaceutical Co., Ltd. *Lactobacillus paracasei* (Lactobacillaceae *L. paracasei* subsp. paracasei): Isolated from yogurt. Culture Medium: MRS Broth Medium (Tryptone 10.0 g/L, Beef Extract 10.0 g/L, Yeast Extract 5.0 g/L, Glucose 20.0 g/L, Sodium Acetate 5.0 g/L, Diammonium Citrate 2.0 g/L, Tween-80 1.0 mL/L, MgSO_4_.7H_2_O 0.58 g/L, MnSO_4_.4H_2_O 0.25 g/L, pH 6.2 ± 0.2). Biochemical Detection Kit: Malondialdehyde (MDA) Content Assay Kit (A003-1-2), purchased from Nanjing Jiancheng Bioengineering Institute. Reverse Transcription Kit (Cat. No.: RR047A) and Fluorescence Quantitative PCR Premix Reagent (Cat. No.: FP215, SuperReal Color SYBR Green) were purchased from Takara Bio Inc. (Japan) and Tiangen Biochemical Technology (Beijing) Co., Ltd., respectively. Other Reagents: Double-distilled water was prepared in-house (purified by Milli-Q ultrapure water system, resistivity ≥18.2 MΩ·cm). Zebrafish culture water was dechlorinated reverse osmosis tap water, supplemented with NaHCO_3_ (final concentration of 0.1 mmol/L) and NaCl (final concentration of 0.5 mmol/L) to adjust water quality parameters.

#### 2.1.2 Main instruments

Biochemical Analysis Equipment: AU5800 Automatic Biochemical Analyzer (Beckman Coulter, United States), used to detect the activities of alanine aminotransferase (ALT) and creatine kinase-MB (CK-MB), and the content of cardiac troponin I (cTnI). Molecular Biology Equipment: StepOnePlus Real-Time PCR System (Applied Biosystems, United States), used for gene expression analysis; Automatic Nucleic Acid Extraction System (Shanghai ZJ Bio-Tech Co., Ltd., China), used for genomic DNA extraction; UV2310II UV-Vis Spectrophotometer (Shanghai Techcomp Scientific Instrument Co., Ltd., China), used for DNA concentration and purity determination. Microbial Culture Equipment: HH.B11.500-S Constant Temperature Incubator (Shanghai Yuejin Medical Instrument Co., Ltd., China), used for *L. paracasei* culture. −80 °C ultra-low temperature freezer (Sartorius, Germany), used for strain cryopreservation. Other Equipment: Synergy2 Microplate Reader (BioTek, United States), used for MDA kit assays; Sigma 3–18 K Refrigerated High-Speed Centrifuge (Sigma, United States, speed ≤18,000 × g, temperature range −4 °C to 40 °C), used for tissue homogenate centrifugation; IX73 Inverted Microscope (Olympus, Japan), used for zebrafish dissection observation; Recirculating Aquaculture System, used for zebrafish rearing, equipped with temperature control (28 °C ± 2 °C), aeration (dissolved oxygen ≥6 mg/L), and filtration modules.

### 2.2 Preparation of experimental materials

#### 2.2.1 Preparation of pueraria lobata extract (PRE)

The specific steps were as follows: Sixty grams of Pueraria lobata was extracted with 600 mL of water (drug-to-solvent ratio 1:10, w/v) using an ultrasonic cleaner (power 150–200 W, temperature 40 °C–50 °C) for 45 min. After extraction, the mixture was centrifuged at 8,000 r/min for 10 min. The collected supernatant was stored at −80 °C until use. The resulting extract was concentrated to a drug-extract ratio (DER) of approximately 5-7:1 (crude drug to dried extract). Puerarin and Daidzin and Daidzein is typically identified as the major active metabolite. The batch used in this study was analyzed by high-performance liquid chromatography (HPLC) according to the methods described in reference (Kang et al., 2019). All measurements were conducted in triplicate. To confirm the taxonomic identity of the *Lactobacillus* paracasei strain used in this study, 16S rRNA gene sequencing was performed. Genomic DNA was extracted from the activated bacterial culture. The 16S rRNA gene was amplified by PCR using universal primers 27F (5’-AGA​GTT​TGA​TCC​TGG​CTC​AG-3’) and 1492R (5’-GGT​TAC​CTT​GTT​ACG​ACT​T-3’). The PCR conditions were as follows: initial denaturation at 96 °C for 150 s; 30 cycles of 96 °C for 30 s, 55 °C for 30 s, and 72 °C for 90 s; and final extension at 72 °C for 7 min. The PCR products were purified and sequenced. The obtained sequences were compared with the NCBI GenBank database using BLAST.2.2.2. Preparation of *L. paracasei* Suspension.

Strain Activation: The strain cryopreserved at −80 °C was streaked onto MRS solid medium (supplemented with 1.5% agar) and anaerobically incubated at 37 °C for 48 h. A single colony was then inoculated into 5 mL of MRS broth medium and incubated at 37 °C with shaking (180 r/min) for 24 h to complete initial activation. Suspension Preparation: The culture was centrifuged at 6,000 × g for 10 min at 4 °C. The supernatant was discarded, and the bacterial pellet was washed twice with sterile physiological saline (0.9% NaCl). The pellet was then resuspended in MRS broth medium containing 10% glycerol, and the viable bacterial concentration was adjusted to 1 × 10^8^ CFU/mL. The suspension was aliquoted and stored at −80 °C. Before use, the frozen bacterial suspension was thawed and mixed with PRE to achieve final concentrations of 1 × 10^6^ CFU/mL for *L. paracasei* and 50 μg/mL for PRE. The mixture was then incubated anaerobically at 37 °C for 24 h to allow fermentation and biotransformation. After this pre-incubation, the resulting Lac_PRE complex was used directly for animal experiments. To verify bacterial viability after the 24 h co-incubation, an aliquot of the complex was serially diluted and plated on MRS agar. Colonies were counted after 48 h of anaerobic incubation at 37 °C; the viable cell count remained consistently at approximately 1 × 10^6^ CFU/mL throughout the experiments.

### 2.3 Experimental animals and housing conditions

#### 2.3.1 Experimental animals

Animal Ethics: This study strictly adhered to the relevant requirements of the Chinese national standard “Experimental animals—General requirements for biosafety of animal experiments” (GB/T 43,051–2023). As adult zebrafish were used in the experiments, ethical approval was obtained from the Ethics Committee of Shenzhen Center for Prevention and Treatment of Occupational Diseases (Approval No.: LL-2024018).

The experimental procedures strictly followed the safety operation regulations stipulated by the Shenzhen Center for Prevention and Treatment of Occupational Diseases. All hazardous waste was properly disposed of. All experiments strictly adhered to Good Laboratory Practice (GLP) guidelines.

Wild-type AB strain zebrafish (*Danio rerio*), with females measuring (32 ± 3) mm in body length and weighing (0.32 ± 0.08) g, and males measuring (28 ± 2) mm in body length and weighing (0.28 ± 0.06) g, were purchased from Shandong Yixiyue Biotechnology Co., Ltd.

#### 2.3.2 Housing conditions

Housing conditions were set with reference to the Organisation for Economic Co-operation and Development (OECD) “Guideline for Testing of Chemicals No. 203: Fish, Acute Toxicity Test” and the Chinese national standard GB/T 13267—1991 “Water quality—Determination of the acute toxicity of substances to freshwater fish (Zebrafish, Brachydanio rerio).”

Aquaculture System: Recirculating water tanks with a single tank volume of 9 L, each housing ≤30 zebrafish.

Water Quality Parameters: Water temperature (28 ± 0.5) °C, pH 7.2 ± 0.2 (adjusted with 1 mol/L HCl or NaOH), conductivity (550 ± 10) μS/cm, dissolved oxygen ≥6 mg/L (continuous aeration), ammonia nitrogen ≤0.1 mg/L.

Lighting and Feeding: Light-dark cycle 14 h:10 h (light on 08:00–22:00). Freshly hatched zebrafish feed was provided daily at 09:00 and 17:00 (feeding amount adjusted to be consumed within 5 min).

Acclimation Period: After purchase, zebrafish were acclimated for 7 days under experimental conditions. During this period, activity, feeding behavior, and mortality were observed daily. Malformed or lethargic individuals were removed. Feeding was stopped 24 h before the experiment.

### 2.4 Experimental design and treatments

#### 2.4.1 Determination of H_2_O_2_ exposure dose (acute toxicity test)

A static renewal method was used for the H_2_O_2_ acute toxicity test to determine the subacute exposure dose.

Grouping: Acclimated zebrafish were randomly divided into 4 groups, 12 fish per group (half male, half female): control group (0 mmol/L H_2_O_2_, i.e., zebrafish culture water), 1 mmol/L H_2_O_2_ group, 2 mmol/L H_2_O_2_ group, and 3.5 mmol/L H_2_O_2_ group.

Exposure Treatment: Each group was placed in a 9 L tank containing the corresponding concentration of H_2_O_2_. The exposure solution was renewed daily. Fish were observed for 96 h.

Observation Parameters: Mortality was recorded every 6 h (death criteria: no gill movement, no swimming response upon gentle touch of the caudal fin). Dead individuals and feces were promptly removed.

Dose Calculation: The 96-h median lethal concentration (96-h LC_50_) of H_2_O_2_ for zebrafish was calculated using the probit method (Bliss method) (Rao et al., 2022). Based on preliminary experimental results, 2.0 mmol/L H_2_O_2_ was selected as the subacute exposure concentration in the formal experiment.

#### 2.4.2 Metabolomic analysis of the complex before and after mixing

To investigate whether biochemical reactions occurred after mixing *Lactobacillus* paracasei and Puerariae Radix extract (PRE), a metabolomic analysis was performed. Three groups were compared: PRE group (PRE alone, 50 μg/mL), L. paracasei group (L. paracasei alone, 1 × 10^6^ CFU/mL), and Lac_PRE group (the mixed complex containing 1 × 10^6^ CFU/mL of L. paracasei and 50 μg/mL of PRE, incubated anaerobically at 37 °C for 24 h). Each group was prepared in triplicate. Samples were analyzed using ultra-high-performance liquid chromatography coupled with a Thermo Fisher Q-Exactive mass spectrometer (UHPLC-Q-Exactive-MS) operating in both negative ion mode (NIM) and positive ion mode (PIM). Data processing, including peak alignment, peak extraction, deconvolution, and identification, was performed using the Progenesis QI platform. Compounds were annotated based on accurate mass, MS/MS fragmentation patterns, and isotope similarity matching using public databases (HMDB and METLIN). Compounds with zero abundance in one group but present in another were considered group-specific by Venn Analysis.

#### 2.4.3 Intervention experiment grouping and treatment

Acclimated zebrafish were randomly divided into 4 groups, 20 fish per group (half male, half female), for a 14-day treatment period. The specific groups were as follows:

Control Group (Con): Zebrafish culture water was renewed daily. At 09:00, after feeding, 5 mL of sterile physiological saline was added.

H_2_O_2_ Exposure Group (H_2_O_2_): Culture water containing 2.0 mmol/L H_2_O_2_ was renewed daily. At 09:00, after feeding, 5 mL of sterile physiological saline was added.

Composite Treatment Group (H_2_O_2__Lac_PRE): Culture water containing 2.0 mmol/L H_2_O_2_ was renewed daily. At 09:00, after feeding, 5 mL of the composite (final concentration: 1 × 10^6^ CFU/mL for *L. paracasei*, 50 μg/mL for PRE) was added.

Composite Control Group (Lac_PRE): Zebrafish culture water was renewed daily. At 09:00, after feeding, 5 mL of the composite was added.

During the treatment period, zebrafish behavioral changes (e.g., swimming speed, schooling behavior) were observed daily, and mortality was recorded. At the end of the 14-day treatment, zebrafish were euthanized using cryoanesthesia (4 °C ice bath for 10 min). Heart, liver, and whole intestine tissues were dissected for subsequent analyses. All experimental procedures, including grouping, drug administration, and endpoint assessments, were performed by investigators blinded to the treatment allocation to minimize bias.

#### 2.4.4 Experimental design schematic

The schematic diagram of the experimental design is shown in Supplementary Figure 1.

### 2.5 Detection parameters and methods

#### 2.5.1 Biochemical indicator detection in heart and liver

Tissue Homogenate Preparation: Heart (pooled sample from 3–4 fish/group) and liver (pooled sample from 3–4 fish/group) tissues from each group were taken, rinsed twice with pre-cooled physiological saline, blotted dry with filter paper, and weighed. Pre-cooled physiological saline was added at a tissue-to-saline ratio of 1:9 (w/v). Tissues were homogenized using a tissue homogenizer on ice (3,000 r/min, 30 s × 3 cycles). The homogenate was centrifuged at 4 °C, 12,000 × g for 15 min. The supernatant (tissue homogenate supernatant) was collected for indicator detection.

Indicator Detection:

ALT, CK-MB, and cTnI: Activities of ALT in liver and CK-MB in heart, as well as the content of cTnI, were detected using the AU5800 biochemical analyzer according to the respective kit instructions.
MDA Content: The thiobarbituric acid (TBA) method was used. Absorbance at 532 nm was measured with a microplate reader to calculate MDA content (nmol/mg prot).
Sample pooling strategy and definition of n: Due to the small size of zebrafish tissues (heart, liver, and whole intestine from individual fish), tissue samples from multiple fish were pooled to obtain sufficient material for each assay. Specifically, for each experimental group, 3–4 fish were randomly assigned to one pool, and three independent pools (each from different fish) were generated per group, yielding three biological replicates per group (n = 3 per group for each endpoint). A total of 9–12 fish were used per group across the three pools. Each pool was processed and analyzed as a single independent unit; thus, all statistical analyses were performed with n = 3 per group, where n refers to the number of independent pooled biological replicates. Results are expressed as median with interquartile range (IQR, P25–P75) due to the small sample size and non-normal distribution of the data, as detailed in the statistical analysis section.

#### 2.5.2 Intestinal microbiota analysis (bacteroidetes and firmicutes abundance, and F/B ratio)

Intestinal Genomic DNA Extraction: Whole intestine tissues from each group (pooled sample from 3–4 fish/group) were taken, and intestinal contents (approximately 100 mg) were collected. Genomic DNA was extracted using a modified CTAB method: Intestinal contents were mixed with 500 μL CTAB extraction buffer (containing 2% CTAB, 100 mmol/L Tris-HCl pH 8.0, 20 mmol/L EDTA, 1.4 mol/L NaCl) and incubated in a 65 °C water bath for 30 min. An equal volume of chloroform-isoamyl alcohol (24:1, v/v) was added, mixed vigorously, and centrifuged at 4 °C, 10,000 × g for 10 min. The supernatant was collected, mixed with 0.8 volumes of isopropanol to precipitate DNA. The DNA pellet was washed twice with 75% ethanol, air-dried, and dissolved in 50 μL TE buffer (containing RNase A, final concentration of 20 μg/mL). The DNA was stored at −20 °C. Consistent with the sample pooling strategy described in Section 2.5.1, three independent pools (each from 3–4 fish) were prepared per group, resulting in n = 3 biological replicates per group for microbiota analysis.

#### 2.5.3 Real-time quantitative PCR (qPCR) detection

Intestine: Using intestinal genomic DNA as template, the relative gene abundance of Bacteroidetes and Firmicutes was detected, with the universal bacterial gene (ALL) as the internal reference gene.

Heart and Liver: Using heart and liver genomic DNA as template, the relative expression levels of COX, TNF-α, and IL-6 genes were detected, with the rps gene as the internal reference gene.

Primer sequences are shown in Table 2. Reaction system (20 μL): 2× SuperReal Color PreMix 10 μL, forward and reverse primers (10 μmol/L) 0.5 μL each, DNA template (1 ng/μL) 4 μL, nuclease-free water 5 μL. Reaction conditions: 95 °C pre-denaturation for 5 min; 40 cycles of 95 °C denaturation for 30 s, annealing at temperature specified in Table 1 for 30 s, and 72 °C extension for 30 s; final extension at 72 °C for 10 min. Melting curve analysis (60 °C–95 °C, increasing 0.5 °C every 10 s) was performed to verify product specificity. The relative abundance of Bacteroidetes and Firmicutes was calculated using the 2^−^ΔΔCt method, and the Firmicutes/Bacteroidetes ratio (F/B ratio) was calculated. Each sample was measured in triplicate. Quality Control for qPCR Analysis: The specificity of the primers for Bacteroidetes and Firmicutes (Table 2) was verified by melting curve analysis (single peak). Three technical replicates were performed for each biological sample, and three biological replicates were analyzed per group. Biological replicates consisted of three independent pooled samples per group (n = 3 per group), as described in Section 2.5.1.

**TABLE 1:** Result for bacterial identification.

Number	Result	Score
1	*Lactobacillus* paracasei	9.277
2	*Lactobacillus* paracasei	9.044
3	*Lactobacillus* paracasei	8.399
4	*Lactobacillus* paracasei	8.289
5	*Lactobacillus* paracasei	8.267
6	*Lactobacillus* paracasei	8.144
7	*Lactobacillus* paracasei subsp.paracasei	7.809
8	*Lactobacillus* paracasei	7.700
9	*Lactobacillus* paracasei	7.536
10	*Lactobacillus* paracasei	7.361

**TABLE 2:** Primer sequences used for qPCR analysis.

Assay	Primer (5′-3′)	Annealing temp (°C)	size (bp) of product
ALL	F: ACT​CCT​ACG​GGA​GGC​AGC​AG	60	126
​	R: ATTACCGCGGCTGCTGG	​	​
*Bacteroidetes*	F: GGARCATGTGGTTTAATTCGATGAT	60	126
​	R: AGCTGACGACAACCATGCAG	​	​
*Firmicutes*	F: GGAGYATGTGGTTTAATTCGAAGCA	60	200
​	R: CAGACGGAGTATTTACGCTCAG	​	​
Cox	F: ACC​AGG​ATT​CGG​CAT​TAT​CTC	60	104
​	R: CTCGGGTGTCTACATCCATTC	​	​
Rps11	F: CTC​TGA​CGA​CAC​TGC​CTT​ATG	60	205
​	R: GAAGATGGTGGGCTGTTTCT	​	​
TNFa	F: AAG​ACC​CAG​GGC​AAT​CAA​CAA​GA	60	261
​	R: GTGCAGCTGATGTGCAAAGACAC	​	​
IL-6	F: GTC​CCC​GTG​TTC​AGC​AGT​AT	60	203
​	R: CTCTGAGTGTCTTCACGTCCTTGT	​	​
Rpl13	F: GAC​AGG​CTG​AAG​GTG​TTT​GAT​G	60	205
​	R: GACTACCATGCGCTTTCTCTTGT	​	​

#### 2.5.4 RT-PCR detection of TNF-α and IL-6

Heart and liver tissues of fish were collected, and total RNA was extracted using TRIzol reagent (Shanghai Sangon Biotech Co., Ltd., Shanghai, China). Reverse transcription-polymerase chain reaction (RT-PCR) was performed following the method described in the previous literature (Xu et al., 2025). Quantitative real-time polymerase chain reaction (qPCR) was conducted in triplicate. The relative expression level of mRNA was calculated using the 2^−ΔΔCt^ method, and the mRNA expression level of ribosomal protein L13 (rpl13) was used as the internal reference for normalization. The sequences of primers used in this study are detailed in Table 2.

### 2.6 Enrichment of functions and prediction of biological mechanism for pueraria and *Lactobacillus* paracasei

Enrichment of functions and signaling pathways of genetic loci with differential functional gene for Pueraria and *Lactobacillus* paracasei was performed based on KOBAS v3.0 http://bioinfo.org/kobas/ using the KEGG database with corrected P-value <0.05. For the construction of biological mechanism prediction diagrams, this study mainly referenced authoritative web databases (https://herbcomb.com/, https://www.imhpc.com/iLABdb).

### 2.7 Statistical analysis

Statistical analysis of all data was performed using IBM SPSS 24.0 software. The data for each group were represented as x ± s or M (P25, P75). For normally distributed and homogenous data, t-test was used for comparison between two groups, one-way analysis of variance (ANOVA) was used for comparison among multiple groups, and Tukey’s test was used for pairwise comparisons between groups. For non-normally distributed or heteroscedastic data, Kruskal–Wallis test was used for comparison among groups.

## 3 Results

### 3.1 Species identification of the target strain

To identify the target strain isolated in this study, matrix-assisted laser desorption/ionization time-of-flight mass spectrometry (MALDI-TOF MS) was employed. This approach involves detecting the characteristic mass spectrum of intracellular protein metabolites of the strain and comparing it against the standard bacterial mass spectrum database.

**FIGURE 1:**
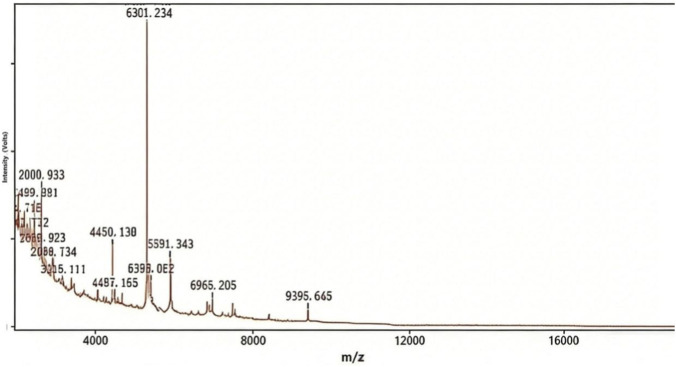
Mass spectrum for bacterial identification.

The characteristic protein mass spectrum of this strain exhibited multiple specific ion peaks with distinct mass-to-charge ratios (m/z) (Figure 1; Table1), such as m/z 6,301.234 and 4,450.138. These characteristic peaks constitute the unique protein fingerprint of the strain, which serves as the core basis for species identification.

After matching this characteristic spectrum with the spectra of standard strains in the database, the results (Table 1) revealed that this strain showed the highest similarity to *L. paracasei* (paracasei), with matching scores ranging from 7.361 to 9.277 (the maximum score reached 9.277). Some matching results could be further refined to *L. paracasei* subsp. paracasei (the paracasei subspecies of L. paracasei), with a corresponding score of 7.809. Within the MALDI-TOF MS identification system, this score range indicates a high degree of homology between the strain and the standard strains in the database, verifying the high reliability of the identification results. The species identification was further confirmed by 16S rRNA gene sequencing (Supplementary Sequence 1). BLAST analysis of the 16S rRNA gene sequence showed the highest similarity (99.49%) to Lacticaseibacillus paracasei ATCC 25302 (formerly *Lactobacillus* paracasei) strains in the NCBI GenBank database. The valid sequence are 973bp by forward primer sequence (Supplementary Figure 5).

### 3.2 Identification of the target strain

By comparing their physicochemical properties and spectral data of HPLC with the corresponding data reported in the literature (Kang et al., 2019), the major compound was unambiguously identified as puerarin (Supplementary Figure 2). The chemical composition of the Puerariae Radix extract (PRE) was characterized by HPLC. A representative chromatogram (Supplementary Figure 2) showed well-resolved peaks corresponding to three major isoflavones. Quantitative analysis revealed that puerarin was the most abundant component, with a content of 2.468 ± 0.119 mg/g of dried extract and a retention time of 5.208 ± 0.010 min, followed by daidzin (0.544 ± 0.020 mg/g, 6.389 ± 0.004 min) and daidzein (0.153 ± 0.007 mg/g, 11.727 ± 0.009 min). All data were derived from three independent injections, with relative standard deviations (RSD) below 2.0% for each component, indicating good repeatability of the analytical method (Supplementary Table 4).

### 3.3 Metabolomic changes after mixing

The TICs of the three experimental groups exhibited markedly distinct chromatographic profiles in both ionization modes, providing initial evidence for substantial compositional differences in the metabolome among PRE, L. paracasei, and Lac_PRE groups (Supplementary Figures 3,4). In the negative ion mode, all three groups displayed dominant signal clusters within the early elution window (0–6 min), a region typically associated with polar and semipolar metabolites including organic acids, phenolic acids, flavonoid glycosides, and nucleotide derivatives. The PRE group exhibited a characteristic dense multi-peak cluster between 0.79 and 5.01 min, with the highest peak at 3.63 min (relative abundance ∼95%), indicating an enrichment of moderately polar secondary metabolites (Supplementary Figure 3a). The L. paracasei group showed a distinctly different profile: the primary apex appeared at 1.36 min (relative abundance ∼95%), and — uniquely compared to the other two groups — a prominent cluster of late-eluting peaks was observed between 11.0 and 14.0 min (Supplementary Figure 3b). The Lac_PRE group exhibited the highest chromatographic complexity, with approximately 16 distinguishable peaks distributed across 0.95–5.87 min and a late single peak at 13.68 min (Supplementary Figure 3c). In the positive ion mode, a salient common feature was observed across all three groups: a dominant high-intensity peak in the 13–14 min region (PRE: 13.76 min; L. paracasei: 13.76 min (Supplementary Figures 4a,b); Lac_PRE: 13.78 min; relative abundance ∼95–100%), along with satellite peaks at 14.00–14.34 min, consistent with hydrophobic lipid-class compounds exhibiting high ionization efficiency in positive mode. The PRE group additionally showed moderate peaks in the polar region (0.79–5.98 min). The L. paracasei group displayed a particularly complex mid-late elution profile with a pronounced series of peaks spanning 9.34–12.93 min. The Lac_PRE group exhibited the broadest early-to-mid elution profile (0.81–6.51 min; 0.81, 1.35, 1.62, 3.25, 3.81, 4.07, 4.33, 4.66, 5.02, 5.46, 5.93, 6.51 min) (Supplementary Figure 4c), reflecting fermentation-derived metabolite enrichment.

Moreover, comprehensive information regarding the identified metabolites—covering metabolite designations, peak area integrals, accurate mass data, unique metabolite serial numbers and molecular formulae—is comprehensively documented in Supplementary Table 1. In this study, a total of 1,350 metabolites were detected in the mixed complex (Lac_PRE group), which was higher than 1,318 metabolites in thePRE group and 1,298 metabolites in the L. paracasei group, indicating enhanced metabolic diversity after co-incubation. Notably, three compounds, including 5-Hydroxy-7,3′,4′-trimethoxy-8-methylisoflavone 5-neohesperidoside,Butyrolactone derivative, Moracin H, were uniquely and stably detected in the Lac_PRE group, with no detectable abundance in both the PRE and L. paracasei groups, confirming the generation of new compounds during co-incubation (Supplementary Material S2). As shown in Supplementary Material S3, significant differences among the three groups were observed for most of the 20 metabolites (P < 0.05). The Lac_PRE group consistently exhibited the highest levels of the majority of compounds, followed by the PRE group, while the L. paracasei group showed the lowest levels.

### 3.4 Acute exposure experiment

This study evaluated the Dunn’s post hoc test with Bonferroni ×2 correction indicated that MDA content in the H_2_O_2_ group (median 2.71 nmol/mg prot, IQR 2.29–4.25) did not differ significantly from either the Con group (median 1.14 nmol/mg prot, IQR 0.70–1.65; adjusted p = 0.175) or the H_2_O_2__Lac_PRE group (median 1.06 nmol/mg prot, IQR 1.00–1.10; adjusted p = 0.175). Descriptively, the H_2_O_2__Lac_PRE group showed a numerically lower median MDA content (61% reduction in median) compared with the H_2_O_2_ group, suggesting a directional attenuation of hepatic effects of hydrogen peroxide (H_2_O_2_) on zebrafish by means of an acute exposure experiment. As shown in the results (Table 3), the mortality of zebrafish exhibited a significant concentration-dependent pattern. After 96 h of exposure, no mortality was observed in the control group (0 mmol/L H_2_O_2_), indicating that the experimental system itself had no significant impact on zebrafish survival. When the H_2_O_2_ concentration increased to 1 mmol/L, the 96-h cumulative mortality was 25.00%; as the concentration rose to 2 mmol/L, the mortality increased to 41.67%; and at an exposure concentration of 3.5 mmol/L, all experimental fish died within 96 h, resulting in a mortality rate of 100%. Based on the above dose-response relationship, the 96-h median lethal concentration (96-h LC_50_) of H_2_O_2_ for the experimental zebrafish was calculated using the linear interpolation method, which was approximately 2.0 mmol/L. A concentration of 2.0 mmol/L was used as the exposure dose in this experiment.

**TABLE 3:** Acute exposure experiment in zebrafish.

Exposure dose (mmol/L)	0–24 h	25–48 h	49–72 h	73–96 h	Mortality rate
0	0	0	0	0	0.00%
1	0	1	1	1	25.00%
2	1	1	2	1	41.67%
3.5	4	2	3	3	100.00%

### 3.5 Liver indices

**FIGURE 2:**
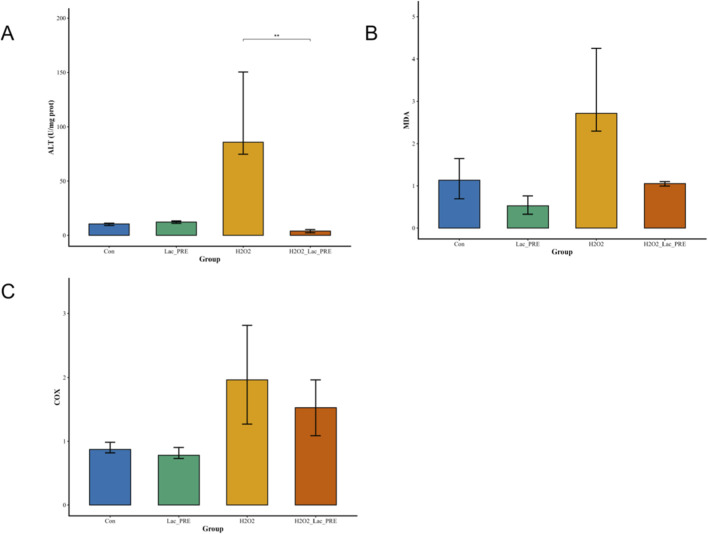
Liver Biochemical Function Indices of Zebrafish after Exposure and Treatment; **(A)** Liver ALT index of zebrafish; **(B)** MDA index; **(C)** *COX* gene index; * indicates P < 0.05, ** indicates P < 0.01. Data are derived from n = 3 biological replicates per group, where each biological replicate represents a pool of tissues from 3–4 individual zebrafish. Boxes represent median with interquartile range; whiskers indicate range. “*P* < 0.05,” **P* <0.01 (Kruskal-Wallis test with Dunn’s *post hoc* test).

To evaluate the toxic effects of hydrogen peroxide (H_2_O_2_) exposure on zebrafish liver and the protective effect of combined intervention with *Lactobacillus* paracasei and PRE, the activity of alanine transaminase (ALT) and the content of malondialdehyde (MDA) in liver tissues were detected. Detailed data are shown in Table 4 and Figure 2.

**TABLE 4:** Biochemical function indices of the liver in zebrafish after exposure and Treatment (Median, P25, P75).

Indicator	Con	H_2_O_2_	H_2_O_2__Lac_PRE	Lac_PRE	H-statistic	p Value
ALT	10.64 (9.14, 11.34)	85.78 (74.79, 150.47)	3.96 (2.39, 5.4)	12.39 (11.18, 13.24)	13.06	0.005
COX	0.87 (0.82, 0.98)	1.96 (1.27, 2.82)	1.52 (1.09, 1.96)	0.78 (0.73, 0.90)	6.51	0.089
MDA	1.14 (0.70, 1.65)	2.71 (2.29, 4.25)	1.06 (1.00, 1.10)	0.53 (0.33, 0.77)	9.77	0.021

Data are presented as median (P25, P75) based on n = 3 biological replicates per group, where each biological replicate represents a pool of tissues from 3–4 individual zebrafish. Therefore, the reported medians and interquartile ranges reflect variation among pooled samples rather than inter-individual variation. The Kruskal-Wallis H test was used to account for the small sample size and non-normally distributed data.

As shown in Table 4, because the data were not normally distributed, a Kruskal–Wallis H test was used to compare ALT activities among the groups. The test revealed a statistically significant difference (H = 13.06, p = 0.005). Subsequently, pairwise comparisons were performed using Dunn’s test with Bonferroni correction. The ALT activity in the H_2_O_2_-exposed group (median 85.78 U/mg prot, IQR 74.79–150.47) was numerically higher than that in the blank control group (Con, median 10.64 U/mg prot, although this difference did not reach statistical significance after Bonferroni correction (adjusted p = 0.075). After combined intervention with *L. paracasei* and PRE, the ALT activity in the H_2_O_2__Lac_PRE group (median 3.96 U/mg prot, IQR 2.39–5.40) was significantly lower than that in the H_2_O_2_ group (adjusted p < 0.01) and showed no significant difference from the Con group (adjusted p > 0.05). The ALT activity in the Lac_PRE group (without H_2_O_2_ exposure, median 12.39 U/mg prot, IQR 11.18–13.24) also did not differ significantly from the Con group (adjusted p > 0.05).

A Kruskal–Wallis H test was used to compare MDA levels among the groups. The test revealed statistically significant difference (H = 9.77, p = 0.021). Dunn’s post hoc test with Bonferroni ×2 correction indicated that MA content in the H_2_O_2_ group (median 2.71 nmol/mg prot, IQR 2.29–4.25) did not differ significantly from either the Con group (median 1.14 nmol/mg prot, IQR 0.70–1.65; adjusted p = 0.175) or the H_2_O_2__Lac_PRE group (median 1.06 nmol/mg prot, IQR 1.00–1.10; adjusted p = 0.175). Descriptively, the H_2_O_2__Lac_PRE group showed a numerically lower median MDA content (61% reduction in median) compared with the H_2_O_2_ group, suggesting a directional attenuation of hepatic.

A Kruskal-Wallis H test was used to compare COX levels among the groups. The test revealed no statistically significant difference (H = 6.51, p = 0.089). Therefore, pairwise comparisons were not performed. Descriptive statistics are as follows: the COX value in the H_2_O_2_ group (median 1.96, IQR 1.27–2.82) was numerically higher than that in the Con group (median 0.87, IQR 0.82–0.98). In the Lac_PRE group (median 0.78, IQR 0.73–0.90), the COX value was close to that in the Con group. The COX value in the H_2_O_2__Lac_PRE group (median 1.53, IQR 1.09–1.96) was numerically lower than that in the H_2_O_2_ group. However, none of these differences reached statistical significance based on the global Kruskal-Wallis test (H = 6.51, p = 0.089).

### 3.6 Heart results

**FIGURE 3:**
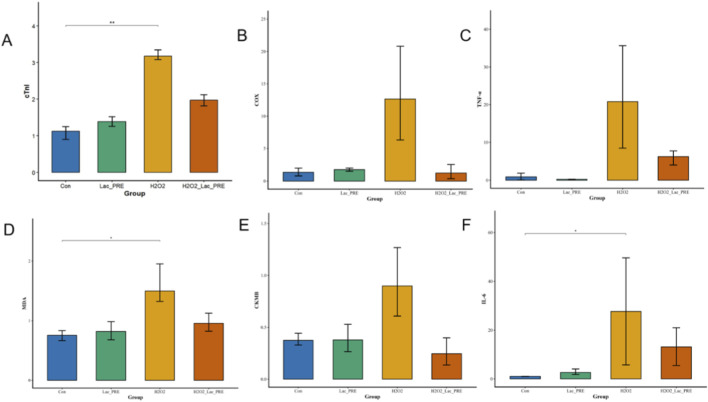
Cardiac Biochemical Function Indices of Zebrafish after Exposure and Treatment; **(A)** Cardiac troponin I (cTnI) index of zebrafish; **(B)** Changes in cardiac *COX* gene expression; **(C)** Tumor necrosis factor-α (TNF-α) index; **(D)** MDA index; **(E)** CK-MB index; **(F)** Interleukin-6 (IL-6) index; * indicates P < 0.05, ** indicates P < 0.01. Data are derived from n = 3 biological replicates per group, where each biological replicate represents a pool of tissues from 3–4 individual zebrafish. Boxes represent median with interquartile range; whiskers indicate range. “*P* < 0.05,” **P* <0.01 (Kruskal-Wallis test with Dunn’s *post hoc* test).

To investigate the toxic effects of hydrogen peroxide (H_2_O_2_) exposure on zebrafish heart, the content of cardiac troponin I (cTnI), creatine kinase-MB (CK-MB), malondialdehyde (MDA), and the expression level of cyclooxygenase (COX), tumor necrosis factor-α (TNF-α), and interleukin-6 (IL-6) in heart tissues were further detected. Detailed data are shown in Table 5; Figures 3A–F.

**TABLE 5:** Biochemical Function Indices of the Heart in Zebrafish after Exposure and Treatment (Median, P25, P75).

Indicator	Con	H_2_O_2_	H_2_O_2__Lac_PRE	Lac_PRE	H-statistic	p Value
cTnI	1.12 (0.90, 1.25)	3.17 (3.08, 3.35)	1.97 (1.82, 2.12)	1.39 (1.26, 1.52)	13.26	0.004
CK-MB	0.38 (0.33, 0.44)	0.90 (0.61, 1.27)	0.25 (0.14, 0.40)	0.38 (0.27, 0.53)	7.08	0.069
MDA	0.76 (0.67, 0.83)	1.50 (1.32, 1.95)	0.96 (0.82, 1.12)	0.82 (0.68, 0.99)	8.6	0.035
COX	1.34 (0.79, 1.99)	12.64 (6.32, 20.82)	1.22 (0.38, 2.54)	1.76 (1.50, 2.00)	8.62	0.035
TNFα	0.90 (0.07, 1.84)	20.81 (8.47, 35.69)	6.25 (4.01, 7.71)	0.25 (0.19, 0.28)	10.81	0.013
IL-6	1.04 (0.97, 1.07)	27.65 (5.75, 49.65)	13.14 (5.47, 21.02)	2.64 (1.82, 4.08)	11.4	0.01

Data are presented as median (P25, P75) based on n = 3 biological replicates per group, where each biological replicate represents a pool of tissues from 3–4 individual zebrafish. Therefore, the reported medians and interquartile ranges reflect variation among pooled samples rather than inter-individual variation. The Kruskal-Wallis H test was used to account for the small sample size and non-normally distributed data.

As shown in Table 5, because the data were not normally distributed, Kruskal–Wallis H tests were used to compare each biochemical index among the five groups (Con, H_2_O_2_, H_2_O_2__Lac_PRE, Lac_PRE). For indices with a statistically significant overall test, pairwise comparisons were subsequently performed using Dunn’s test with Bonferroni correction.

For cardiac troponin I (cTnI), the Kruskal-Wallis test revealed a significant difference among groups (H = 13.26, p = 0.004). The cTnI level in the H_2_O_2_ group (median 3.17, IQR 3.08–3.35) was significantly higher than that in the Con group (median 1.12, IQR 0.90–1.25; adjusted p < 0.01). The H_2_O_2__Lac_PRE group (median 1.97, IQR 1.82–2.12) showed a numerically lower cTnI level compared with the H_2_O_2_ group (38% reduction in median), but this difference did not reach statistical significance after Bonferroni correction (adjusted p = 0.470).

For CK-MB, the Kruskal-Wallis test showed no statistically significant difference among the groups (H = 7.08, p = 0.069). Therefore, pairwise comparisons were not performed. Descriptive statistics are as follows: CK-MB level in the H_2_O_2_ group (median 0.90, IQR 0.61–1.27) was higher than that in the Con group (median 0.38, IQR 0.33–0.44); the H_2_O_2__Lac_PRE group (median 0.25, IQR 0.14–0.40) was lower than the H_2_O_2_ group; and the Lac_PRE group (median 0.38, IQR 0.27–0.53) was similar to the Con group. However, none of these differences reached statistical significance based on the global test.

For MDA, a significant overall difference was found (H = 8.60, p = 0.035). The MDA content in the H_2_O_2_ group (median 1.50, IQR 1.32–1.95) was significantly higher than that in the Con group (median 0.76, IQR 0.67–0.83; adjusted p < 0.05). The H_2_O_2__Lac_PRE group (median 0.96, IQR 0.82–1.12) showed a numerically lower MDA level compared with the H_2_O_2_ group (36% reduction in median), but this difference was not statistically significant after Bonferroni correction (adjusted p = 0.149).

For COX, the Kruskal-Wallis test also indicated a significant difference (H = 8.62, p = 0.035). The COX level in the H_2_O_2_ group (median 12.64, IQR 6.32–20.82) was significantly higher than that in the Con group (median 1.34, IQR 0.79–1.99; adjusted p = 0.029). The H_2_O_2__Lac_PRE group (median 1.22, IQR 0.38–2.54) showed a significantly lower COX level compared with the H_2_O_2_ group (adjusted p = 0.023).

For TNFα, a significant overall difference was observed (H = 10.81, p = 0.013). The TNFα level in the H_2_O_2_ group (median 20.81, IQR 8.47–35.69) was significantly higher than that in the Con group (median 0.90, IQR 0.07–1.84; adjusted p = 0.023). The H_2_O_2__Lac_PRE group (median 6.25, IQR 4.01–7.71) showed a markedly lower median TNF-α level compared with the H_2_O_2_ group (70% reduction in median); however, due to high within-group variability in the H_2_O_2_ group, this difference did not reach statistical significance after Bonferroni correction (adjusted p = 0.746).

For IL-6, the Kruskal-Wallis test revealed a significant difference (H = 11.40, p = 0.010). The IL-6 level in the H_2_O_2_ group (median 27.65, IQR 5.75–49.65) was significantly higher than that in the Con group (median 1.04, IQR 0.97–1.07; adjusted p < 0.01). The H_2_O_2__Lac_PRE group (median 13.14, IQR 5.47–21.02) showed a numerically lower median IL-6 level compared with the H_2_O_2_ group (52% reduction), but this difference was not statistically significant (adjusted p = 1.000), likely reflecting the wide inter-individual variability in the H_2_O_2_ group (IQR 5.75–49.65).

### 3.7 Intestinal results

**FIGURE 4:**
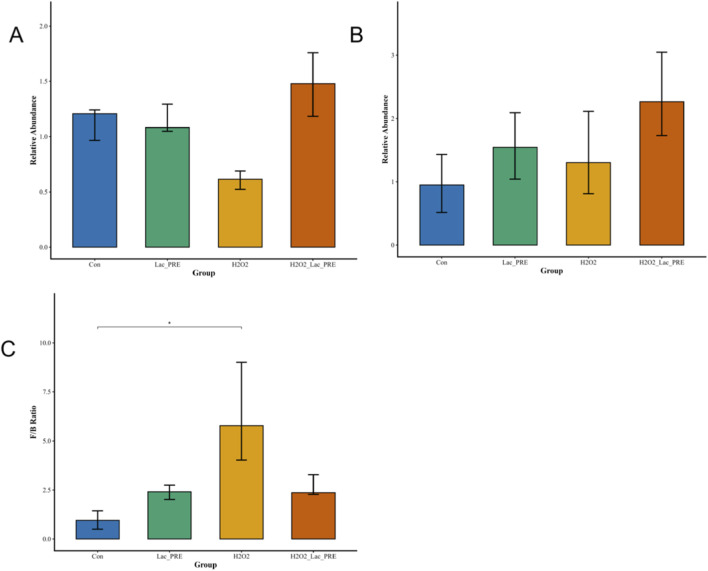
Intestinal Microbiota Indices of Zebrafish after Exposure and Treatment; **(A)** Relative abundance of *Bacteroides*; **(B)** Relative abundance of *Firmicutes*; **(C)** Firmicutes/*Bacteroides* Ratio (F/B Ratio, F: *Firmicutes*, B: *Bacteroides*); * indicates P < 0.05, ** indicates P < 0.01. Data are derived from n = 3 biological replicates per group, where each biological replicate represents a pool of tissues from 3–4 individual zebrafish. Boxes represent median with interquartile range; whiskers indicate range. “*P* < 0.05,” **P* <0.01 (Kruskal-Wallis test with Dunn’s *post hoc* test).

To investigate the effects of H_2_O_2_ exposure and combined intervention on the intestinal microecology of zebrafish, the relative abundances of Bacteroidetes (phylum) and Firmicutes (phylum) in intestinal contents, as well as their ratio (F/B Ratio), were detected. Detailed data are shown in Table 6; Figures 4A–C.

**TABLE 6:** Changes in intestinal microbiota of zebrafish after exposure and Treatment (Median, P25, P75).

Indicator	Con	H_2_O_2_	H_2_O_2__Lac_PRE	Lac_PRE	H statistic	p Value
*Bacteroides*	1.21 (0.97, 1.24)	0.62 (0.52, 0.69)	1.48 (1.18, 1.76)	1.08 (1.05, 1.29)	6.79	0.079
Firmicutes	0.95 (0.52, 1.43)	1.30 (0.81, 2.11)	2.27 (1.73, 3.05)	1.55 (1.04, 2.09)	4.41	0.22
F/B Ratio	0.95 (0.50, 1.44)	5.78 (4.02, 9.01)	2.36 (2.28, 3.28)	2.41 (2.01, 2.75)	8.36	0.039

Data are presented as median (P25, P75) based on n = 3 biological replicates per group, where each biological replicate represents a pool of tissues from 3–4 individual zebrafish. Therefore, the reported medians and interquartile ranges reflect variation among pooled samples rather than inter-individual variation. The Kruskal-Wallis H test was used to account for the small sample size and non-normally distributed data.

As shown in Table 6, because the data were not normally distributed, Kruskal–Wallis H tests were used to compare each intestinal microbiota indicator among the four groups (Con, H_2_O_2_, H_2_O_2__Lac_PRE, Lac_PRE). For indices with a statistically significant overall test, pairwise comparisons were subsequently performed using Dunn’s test with Bonferroni correction.

For *Bacteroides*, the Kruskal-Wallis test revealed no statistically significant difference among the groups (H = 6.79, p = 0.079). Therefore, pairwise comparisons were not performed. Descriptive statistics are as follows: the *Bacteroides* abundance in the H_2_O_2_ group (median 0.62, IQR 0.52–0.69) was lower than that in the Con group (median 1.21, IQR 0.97–1.24); the H_2_O_2__Lac_PRE group (median 1.48, IQR 1.18–1.76) showed a higher abundance than the H_2_O_2_ group; and the Lac_PRE group (median 1.08, IQR 1.05–1.29) was similar to the Con group. However, none of these differences reached statistical significance based on the global test.

For Firmicutes, the Kruskal-Wallis test also indicated no statistically significant difference (H = 4.41, p = 0.22). The Firmicutes abundance in the H_2_O_2_ group (median 1.30, IQR 0.81–2.11) was slightly higher than that in the Con group (median 0.95, IQR 0.52–1.43); the H_2_O_2__Lac_PRE group (median 2.27, IQR 1.73–3.05) and the Lac_PRE group (median 1.55, IQR 1.04–2.09) also showed higher abundances, but these differences were not statistically significant.

For the F/B ratio (Firmicutes/*Bacteroides* ratio), the Kruskal-Wallis test revealed a statistically significant difference among the groups (H = 8.36, p = 0.039). Subsequently, pairwise comparisons were performed using Dunn’s test with Bonferroni correction. The F/B ratio in the H_2_O_2_ group (median 5.78, IQR 4.02–9.01) was significantly higher than that in the Con group (median 0.95, IQR 0.50–1.44; adjusted p < 0.05). The H_2_O_2__Lac_PRE group (median 2.36, IQR 2.28–3.28) showed a numerically lower F/B ratio compared with the H_2_O_2_ group (59% reduction in median), approaching Con group levels, but this difference was not statistically significant (adjusted p = 1.000).

### 3.8 KEGG pathway analysis and prediction of biological mechanism

**FIGURE 5:**
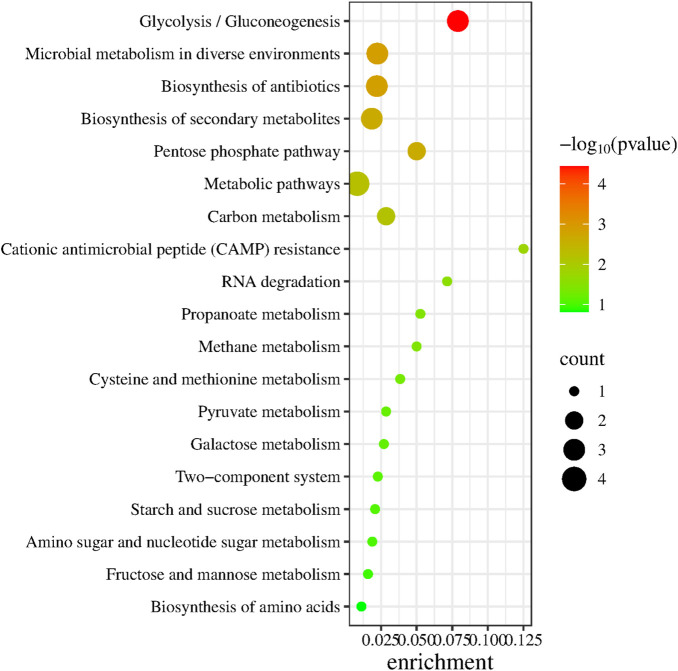
Pathway enrichment of the identified Pueraria genes. Fold enrichment is calculated by dividing gene ratio (the number of genes enriched in the pathway divided by the total number of analyzed genes) with background ratio in KEGG database. P-values were adjusted.

**FIGURE 6:**
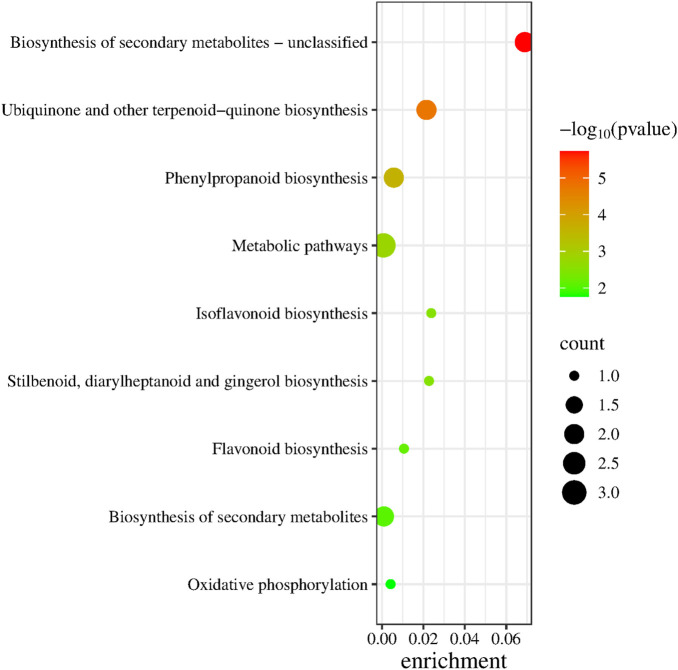
Pathway enrichment of the identified *Lactobacillus paracasei* genes. Fold enrichment is calculated by dividing gene ratio (the number of genes enriched in the pathway divided by the total number of analyzed genes) with background ratio in KEGG database. P-values were adjusted.

**FIGURE 7:**
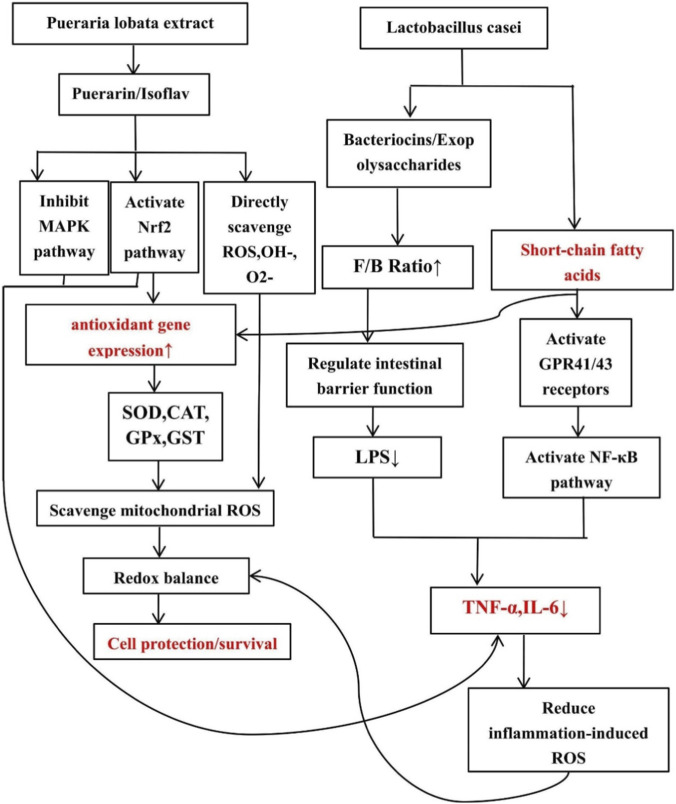
Predicted Synergistic Action Pathways of *Lactobacillus* paracasei and Pueraria lobata Based on Biological Mechanisms. P. lobata extract enhances antioxidant capacity by modulating signaling pathways and directly scavenging reactive oxygen species (ROS). L. casei regulates intestinal barrier function and suppresses pro-inflammatory cytokines via its metabolites. The two agents exert coordinated effects in upregulating antioxidant gene expression and downregulating pro-inflammatory cytokines, ultimately collaboratively maintaining redox homeostasis and conferring cellular protection.

KEGG pathway analysis of Pueraria functional gene and *Lactobacillus* paracasei gene revealed a significant correlation among these gene and microbial metabolism in diverse enviroment pathways and oxidative phosphorylation (Figures 5, 6). According to publicly available data from the website database, the predicted pathways of action based on the biological mechanism are as follows. Pueraria lobata extract (containing puerarin/isoflavones) and *Lactobacillus* paracasei work synergistically to protect intestinal health through integrated antioxidant and anti-inflammatory mechanisms. They directly scavenge reactive oxygen species (ROS) such as hydroxyl radicals and superoxide anions in the gut, mitigating oxidative damage. Furthermore, they enhance endogenous antioxidant defenses by significantly increasing the activities of key enzymes—SOD, CAT, GPx, and GST—and activate the Nrf2 pathway to upregulate downstream antioxidant gene expression. They also protect mitochondrial function by eliminating mitochondrial ROS. In parallel, the combination reduces intestinal levels of the pro-inflammatory cytokines TNF-α and IL-6, and lowers LPS endotoxin content, thereby alleviating inflammation and barrier damage. It modulates inflammatory signaling by inhibiting the MAPK pathway and regulating the NF-κB pathway. Additionally, via activation of GPR41/43 receptors on intestinal epithelial cells, it mediates beneficial effects linked to short-chain fatty acids. Bacteriocins or exopolysaccharides from L. casei further synergize with the botanical extract to strengthen barrier function and modulate flora. Collectively, these actions restore intestinal barrier integrity, regulate gut microbiota by promoting a balanced Firmicutes/Bacteroidetes ratio and inhibiting H_2_O_2_-induced dysbiosis, and enhance epithelial cell survival. Crucially, they break the vicious cycle between oxidative stress and inflammation by scavenging inflammation-derived ROS, resulting in comprehensive protection of the intestinal environment (Figure 7).

## 4 Discussion

### 4.1 H_2_O_2_-induced oxidative damage in zebrafish: a validated multi-organ model

Oxidative stress, as a core pathological hub linking environmental exposure and chronic diseases, essentially refers to a dynamic imbalance caused by the intracellular reactive oxygen species (ROS) production rate exceeding the scavenging capacity (Sule et al., 2025). In this study, hydrogen peroxide (H_2_O_2_) was used as an exogenous oxidative stress inducer, and zebrafish (sharing 87% genetic homology with humans) were employed to establish a multi-organ inflammation model. Its core advantage lies in the ability to simulate the key pathological features of human oxidative stress-related diseases—which is fully validated by the results of this study (Sapeta-Nowinska et al., 2025).

### 4.2 Protective effects of Lac_PRE on hepatic and cardiac function

From the perspective of pathological mechanisms, although H_2_O_2_ itself has limited oxidative activity, it can generate highly reactive hydroxyl radicals (・OH) through the Fenton reaction. The attack of this radical on biological macromolecules is characterized by “multi-target and high destructiveness”: In the heart, ・OH attacks the mitochondrial electron transport chain, inhibits the activity of Complex I/III, triggers an energy metabolism crisis, and further leads to the destruction of myocardial cell membrane integrity (manifested by the release of myocardial-specific marker cTnI) and calcium homeostasis imbalance. Meanwhile, it activates the Cytochrome c Oxidase (COX)-mediated inflammatory cascade, forming a vicious cycle of “oxidative damage - energy deficiency - aggravated inflammation” (Prabu et al., 2006). Ultimately, this resulted in a significant leakage of troponin I (cTnI) in zebrafish myocardial tissue, reflecting the destruction of myocardial cell membrane integrity; a sharp increase in liver ALT activity, indicating hepatocyte necrosis or increased membrane permeability induced by oxidative stress; and a sudden rise in the Firmicutes/*Bacteroides* ratio (F/B ratio) in intestinal microbiota, which is consistent with the classic feature of oxidative stress-induced intestinal microecological imbalance (Fouda et al., 2024). These pathological changes are highly consistent with the molecular mechanisms of human atherosclerosis (myocardial oxidative damage), non-alcoholic fatty liver disease (hepatocyte oxidative stress), and inflammatory bowel disease (intestinal microbiota dysbiosis), providing a reliable model basis for the subsequent evaluation of intervention effects (Alexandrescu et al., 2024).

Pueraria lobata, as a food-medicinal plant, has been confirmed in multiple studies to exhibit antioxidant effects through its core active metabolite puerarin (Ruan et al., 2025). This study further revealed its specific molecular pathways in multi-organ protection: On one hand, puerarin can upregulate the expression of endogenous antioxidant enzymes such as superoxide dismutase—which is consistent with the conclusion by Wang et al. (Wang et al., 2020) that “puerarin enhances antioxidant enzyme activity by activating the nuclear factor erythroid 2-related factor 2 (Nrf2)/antioxidant response element (ARE) pathway”. However, the application of puerarin extract (PRE) alone has obvious limitations: Although the glucosyl structure of puerarin enhances its water solubility, it reduces its transmembrane absorption efficiency. Previous studies have shown that the bioavailability of puerarin in rats is only approximately 7.0%, and its targets are concentrated in antioxidant and anti-inflammatory pathways, failing to regulate intestinal microecology (Anukunwithaya et al., 2018).

Malondialdehyde (MDA) is a product of lipid peroxidation in the body, which indirectly reflects the degree of peroxidation (Bergin et al., 2021). In addition, excessive ROS can inactivate superoxide dismutase (SOD), thereby losing its protective effect on tissues. Experiments showed that the above indicators changed more significantly in the H_2_O_2_-exposed group: the MDA levels in the heart and liver of the H_2_O_2_-exposed group were the highest. After treating zebrafish with puerarin and lactobacilli, the oxidative stress markers were significantly improved, and a significant difference was observed in the decrease in MDA content suggesting a potential baseline antioxidant effect of the composite even in the absence of oxidative challenge.

### 4.3 Anti-inflammatory mechanisms: regulation of COX, TNF-α, and IL-6 expression

The cyclooxygenase (COX) encoded by the *COX* gene is a key rate-limiting enzyme in the synthesis of prostaglandins (PGs). Its activity level directly affects the production of prostaglandins in the body, thereby regulating pathophysiological processes such as inflammatory response, cell proliferation, and tumorigenesis. COX-2 expression is significantly upregulated (up to 10–80 times the normal level) under stimuli such as inflammation and tumors, promoting the synthesis of inflammatory mediators such as prostaglandin E2 (PGE2) and exacerbating tissue damage and disease progression (Wang and Dubois, 2010). This study found that *COX* gene expression increased after zebrafish were exposed to H_2_O_2_, indicating the occurrence of oxidative damage in the body; oxidative stress can directly activate the *COX* gene. However, after combined treatment with puerarin and lactobacilli, *COX* gene expression decreased. Studies have shown that the isoflavone structure of puerarin can directly inhibit COX activity by competitively binding to the active site of COX-2 and downregulating the NF-κB signaling pathway to reduce COX-2 transcription (Hu et al., 2011). In addition, the composite reduces the induction of the *COX* gene by ROS by decreasing MDA and restoring SOD activity, forming a negative feedback loop of “oxidative stress - COX - inflammation.”

Cardiac cTnI, due to its myocardial specificity, can sensitively detect minor myocardial damage, while CK-MB reflects energy metabolism disorders. After H_2_O_2_ exposure, both increased simultaneously, suggesting that ROS disrupt myocardial cell calcium homeostasis and mitochondrial electron transport chain, triggering myofibril degradation and energy crisis. The liver was damaged by H_2_O_2_, leading to liver cell membrane damage and ALT release into the blood. Previous studies have found that H_2_O_2_ inhibits the activity of Complex I/III, resulting in a 50%–70% reduction in hepatocyte ATP synthesis and the failure of the membrane Na^+^/K^+^-ATPase. Meanwhile, myocardial cell calcium overload (cytoplasmic Ca^2+^ concentration increased by 3-5 folds) and this energy metabolism collapse can explain why CK-MB (myocardial energy marker) and ALT (hepatocyte integrity marker) showed a synergistic increase trend.

### 4.4 Gut microbiota modulation: restoration of F/B ratio homeostasis

A large number of studies have shown that excessive ROS production can not only directly attack cells in organs such as the heart and liver, leading to the release of specific damage markers (e.g., cardiac cTnI/CK-MB, liver ALT), but also disrupt the balance of intestinal microecology, mainly manifested by a significant increase in the Firmicutes/*Bacteroides* ratio (F/B ratio) (Wu et al., 2025). The F/B ratio has been widely recognized as a key indicator for evaluating intestinal microbiota dysbiosis and systemic inflammatory status. The results of this study showed that H_2_O_2_ exposure induced numerical shifts in intestinal microbiota composition directionally consistent with dysbiosis: the relative abundance of Bacteroides numerically decreased (Kruskal-Wallis p = 0.079) and the relative abundance of Firmicutes numerically increased (Kruskal-Wallis p = 0.22), although neither change reached statistical significance. This finding is consistent with previous research conclusions in mammalian models, confirming the inhibitory effect of oxidative stress on beneficial intestinal bacteria (e.g., *Bacteroides*) and the promoting effect on conditional pathogens (many *Firmicutes*).

### 4.5 Proposed synergistic mechanisms of the probiotic-botanical combination

The combined intervention of puerarin and *L. paracasei* (H_2_O_2__Lac_PRE group) exhibited an excellent synergistic protective effect. This treatment not only significantly reversed the abnormal increase in cardiac and liver function markers (ALT, cTnI, CK-MB) but also effectively remodeled the intestinal microbiota structure indicating that Lac_PRE treatment not only prevented damage but may have optimized liver and myocardial energy homeostasis. For hepatic protection, our finding that Lac_PRE treatment reversed H_2_O_2_-induced ALT elevation aligns with a previous study by Zheng et al., who reported that puerarin alone reduced ALT levels in a rat model of liver injury. However, our study demonstrates that the combination with L. paracasei achieves comparable protection at a lower effective dose, suggesting a synergistic effect. Similarly, the reduction in cardiac MDA content observed in our H_2_O_2__Lac_PRE group corroborates the findings of Ding et al. (Puerarin protects against myocardial ischemia/reperfusion injury by inhibiting ferroptosis.), who showed that puerarin protected against myocardial ischemia-reperfusion injury by inhibiting ferroptosis. Oxidative stress induced by H_2_O_2_ can strongly activate the inflammatory response, which is consistent with the literature report that H_2_O_2_ stimulates macrophages to produce TNF-α by activating the p38 MAPK and JNK pathways. The median TNF-α level in the H_2_O_2__Lac_PRE group (6.25) was lower than that in the H2O2 group (20.81), but the difference was not statistically significant (adjusted p = 0.746); pretreatment with puerarin + lactobacilli can effectively inhibit the inflammatory response under basal conditions, indicating its good anti-inflammatory properties, which may be achieved by inhibiting the NF-κB pathway (Yang et al., 2023).

Oxidative stress strongly induces IL-6 expression and promotes the inflammatory cascade by activating the NF-κB pathway, which is consistent with the theory of “inflammation-oxidative stress vicious cycle” induced by H_2_O_2_ (Didion, 2017). The protective effect of puerarin + lactobacilli pretreatment on IL-6 was limited, indicating differences in the inflammatory regulation mechanisms between the two, suggesting that pretreatment may have a selective anti-inflammatory effect. Isoflavones in puerarin and metabolites of lactobacilli can inhibit NF-κB activation and block the transcription of inflammatory factors such as TNF-α and IL-6 (Sun et al., 2024). Our pathway enrichment analysis revealed significant overlap between the genes associated with Pueraria lobata and *Lactobacillus* paracasei and pathways involved in oxidative phosphorylation and microbial metabolism, both of which are closely linked to the mechanism of antioxidant damage.

### 4.6 Metabolomic evidence for biotransformation and enhanced bioactivity of the *L. paracasei*–PRE complex

Co-incubation after mixing facilitates fermentation and biotransformation. Notably, the complex used in this study was prepared via L. paracasei-mediated anaerobic fermentation biotransformation, rather than simple physical mixing of the two components. The metabolomic analysis revealed that co-incubation of L. paracasei with Puerariae Radix extract (PRE) not only increased overall metabolite diversity but also generated unique compounds absent in either component alone. Specifically, the Lac_PRE group contained 1,350 metabolites, exceeding those in the PRE (1,318) and L. paracasei (1,298) groups, indicating a synergistic expansion of the chemical space through microbial fermentation. Notably, three compounds—5-Hydroxy-7,3′,4′-trimethoxy-8-methylisoflavone 5-neohesperidoside, a butyrolactone derivative, and Moracin H—were exclusively detected in the Lac_PRE group. The appearance of these novel metabolites suggests that L. paracasei mediates biotransformation reactions such as deglycosylation, hydroxylation, or esterification, which are known to occur during lactic acid bacterial fermentation. For instance, butyrolactone derivatives are often linked to quorum sensing or secondary metabolism (Zhu et al., 2024), while Moracin H is a phenolic compound with reported antioxidant activity. Furthermore, the Lac_PRE group consistently exhibited the highest levels of the majority of quantified metabolites, including known bioactive isoflavones (e.g., daidzin, puerarin, vitexin). This elevation may result from enhanced extraction or release of plant-derived compounds due to bacterial enzymatic activity (e.g., cellulase, pectinase), or from *de novo* synthesis by L. paracasei under the selective pressure of PRE components.

These metabolomic findings provide a chemical foundation for the observed superior protective effects of Lac_PRE against H_2_O_2_-induced oxidative damage. The increased abundance of antioxidant metabolites and the generation of novel bioactive compounds likely contribute to the enhanced anti-inflammatory and gut microbiota-modulating activities described in this study. Collectively, the metabolomic data reveal that co-incubation of L. paracasei and PRE triggers three interrelated mechanisms: (i) activation of fatty acid β-oxidation (indicated by acylcarnitines), (ii) biotransformation of dietary glycosides into more bioavailable aglycones (e.g., daidzein), and (iii) *de novo* synthesis of anti-inflammatory lipopeptides (e.g., surfactin A). These chemical changes provide a direct mechanistic explanation for the observed protective effects of Lac_PRE against H_2_O_2_-induced oxidative damage in zebrafish, including reduced hepatic ALT and cardiac cTnI levels, decreased MDA content, and restoration of the Firmicutes/Bacteroidetes ratio. Our findings establish a solid chemical foundation for the synergistic antioxidant and anti-inflammatory actions of the L. paracasei–PRE complex, supporting its potential as a natural intervention for oxidative stress-related diseases.

### 4.7 Limitations and future directions

Although this study has achieved phased results, it still has certain limitations: First, A key limitation is that zebrafish absorb compounds via gills and skin, bypassing mammalian gastrointestinal metabolism, which complicates the direct translation of effective doses. Furthermore, despite the conserved F/B ratio, the marked species-specific differences in gut microbiota composition limit the extrapolation of our microbial findings to humans. Second, this study is a short-term intervention; the preventive and improvement effects of long-term intervention on oxidative stress-related chronic diseases (e.g., atherosclerosis, type 2 diabetes) still need to be explored. Third, the discussion on the synergistic mechanism of the composite remains at the “pathway association” level, and the direct interaction between probiotic metabolites and active metabolites of puerarin (e.g., whether new active substances are formed) has not been analyzed in depth.

A key methodological limitation of this study concerns the sample pooling strategy employed for biochemical and microbiota analyses. Due to the small size of zebrafish tissues (e.g., heart, liver, and whole intestine from individual fish), pooled samples from 3–4 fish were used as a single biological replicate, with three such replicates per group (n = 3 per group for each assay). While this pooling approach is common practice in zebrafish research to obtain sufficient tissue mass for endpoint measurements, it has important implications for statistical inference. First, the use of pooled samples reduces the effective sample size to n = 3 per group, limiting the statistical power to detect modest effect sizes and increasing the risk of Type II errors (false negatives). Second, the variance estimates derived from pooled samples reflect inter-pool variability rather than true inter-individual variability, which may underestimate biological variation. Consequently, non-significant results (e.g., for hepatic COX expression, cardiac CK-MB, or intestinal *Bacteroides* and Firmicutes abundances in this study) should be interpreted with caution, as they may reflect insufficient statistical power rather than genuine absence of biological effects. Third, while each pooled sample contained 3–4 fish, the absence of individual-level data precludes assessment of within-group heterogeneity. Therefore, the findings presented here should be considered exploratory and hypothesis-generating, warranting validation in future studies with larger sample sizes and, where feasible, individual-level analyses.

Despite these limitations, our findings have important translational implications. The demonstration that a probiotic-botanical drug combination can simultaneously protect multiple organs and modulate gut microbiota suggests a promising strategy for developing functional foods or dietary supplements targeting oxidative stress-related conditions. The use of Pueraria lobata, a plant with established food and medicinal use, combined with L. paracasei, a common probiotic strain, provides a favorable safety profile that facilitates translation. Future research can be advanced from three aspects: First, establish mammalian models more similar to human diseases to evaluate the long-term intervention effect and safety of the composite. Second, use multi-omics techniques (e.g., metabolomics, metagenomics) to analyze the effects of the composite on intestinal microbiota metabolites and the body’s oxidative metabolism pathways, and identify key regulatory targets. Third, optimize the dosage form of the composite to further improve its bioavailability and stability, laying a foundation for clinical transformation and industrial application. In light of these limitations, the conclusions drawn from this study should be regarded as preliminary. The statistically significant findings—particularly for hepatic ALT, cardiac cTnI, cardiac MDA, cardiac TNF-α, and the intestinal F/B ratio—demonstrate proof-of-concept efficacy of the Lac_PRE composite, but the effect sizes and p-values may be imprecise given the small number of pooled replicates. Nonsignificant results (e.g., for hepatic COX, cardiac CK-MB, and intestinal *Bacteroides*/*Firmicutes* abundances) should not be interpreted as evidence of no effect. Future studies with larger sample sizes (e.g., n ≥ 6 biological replicates per group, ideally with individual-level analyses) are needed to confirm the observed trends and to detect smaller effect sizes that may have been missed in this exploratory investigation.

## 5 Conclusion

In conclusion, this exploratory study provides preliminary evidence that the composite of *L. paracasei* and *Pueraria lobata* extract attenuates H_2_O_2_-induced multi-organ oxidative inflammatory damage in zebrafish, with effects observed across hepatic, cardiac, and intestinal endpoints. The findings suggest a potential synergistic mechanism involving antioxidant, anti-inflammatory, and gut microbiota-modulating activities. However, due to the methodological limitations inherent to the sample pooling strategy (effective n = 3 per group for most assays), these results should be interpreted as hypothesis-generating rather than confirmatory. The observed effects, while statistically significant for several key endpoints (e.g., ALT, cTnI, cardiac MDA, cardiac TNF-α, and F/B ratio), require independent replication in studies designed with adequate statistical power and individual-level biological replicates. Nevertheless, this study provides a proof-of-concept foundation for the ‘probiotic-phytochemical’ synergistic approach and offers a reference framework for future investigations into gut-microbiota-targeted interventions against oxidative stress-related pathologies.

## Data Availability

The original contributions presented in the study are included in the article/Supplementary Material; further inquiries can be directed to the corresponding author. The 16S rRNA sequence datasets generated in this study are publicly available here: https://www.ncbi.nlm.nih.gov/nuccore/PZ359233.1/.

## Supplementary material

SUPPLEMENTARY FIGURE S1 An acute toxicity test and a subsequent 14-day intervention experiment. In the acute toxicity test, a mortality assay over 4 days using a hydrogen peroxide (H_2_O_2_) concentration gradient (0, 1, 2, and 3.5 mmol/L) was conducted to determine the sublethal exposure dose of 2.0 mmol/L for the main study. In the chronic experiment, fish were assigned to a control group, a group exposed to 2.0 mmol/L H_2_O_2_, and a group exposed to H_2_O_2_ with prophylactic *Lactobacillus* treatment. After 14 days of daily solution renewal, samples were collected to assess hepatotoxicity (ALT, MDA, COX), cardiotoxicity (cTnI, CK-MB, MDA, COX, TNF-α, IL-6), and gut microbiota composition (relative abundance of Bacteroidetes and Firmicutes, and their ratio), aiming to evaluate the protective effects of *Lactobacillus* against H_2_O_2_-induced oxidative stress and inflammation.

SUPPLEMENTARY FIGURE S2 HPLC chromatograms of **(a)** a standard mixture and **(b)** the Pueraria lobata extract, detected at 254 nm.

SUPPLEMENTARY FIGURE S3 Representative total ion chromatograms (TICs) of the PRE, L. paracasei, and Lac_PRE groups acquired in negative ion mode.

SUPPLEMENTARY FIGURE S4 Representative total ion chromatograms (TICs) of the PRE, L. paracasei, and Lac_PRE groups acquired in positive ion mode.

SUPPLEMENTARY FIGURE S5 Sanger sequencing trace of the 16S rRNA gene. Arrows indicate the boundaries of the valid sequence region selected for downstream analysis.
