# Supplementary figures and images for: Ameliorative effects of a Lactobacillus paracasei and Puerariae Radix extract complex on hydrogen peroxide-induced oxidative damage in zebrafish
Source: Front Pharmacol. 2026 Jun 17;17:1787487. doi: 10.3389/fphar.2026.1787487 (PMC13318986; doi:10.3389/fphar.2026.1787487)

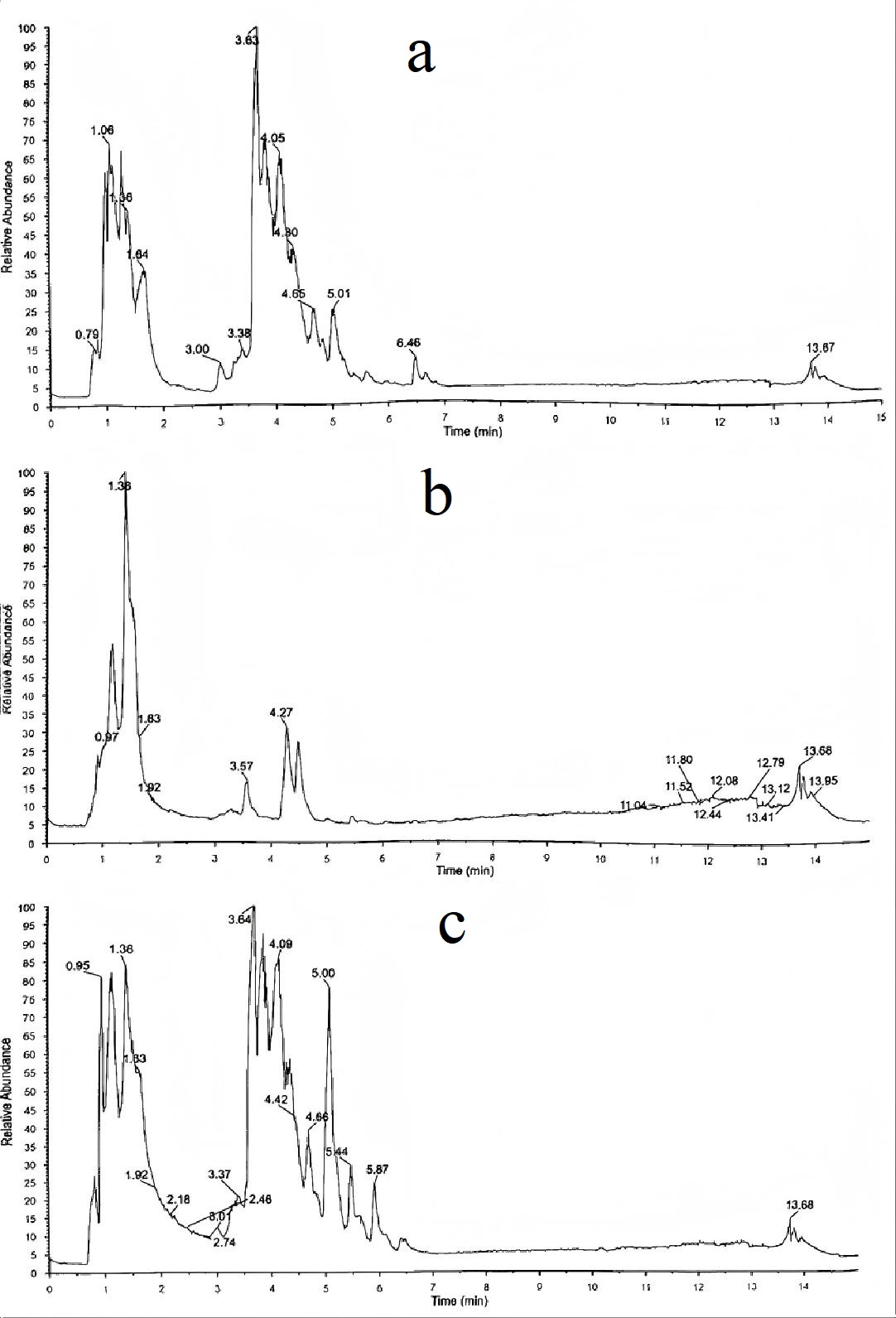

Supplement: Supplementary file 1 [file Image3.jpeg]

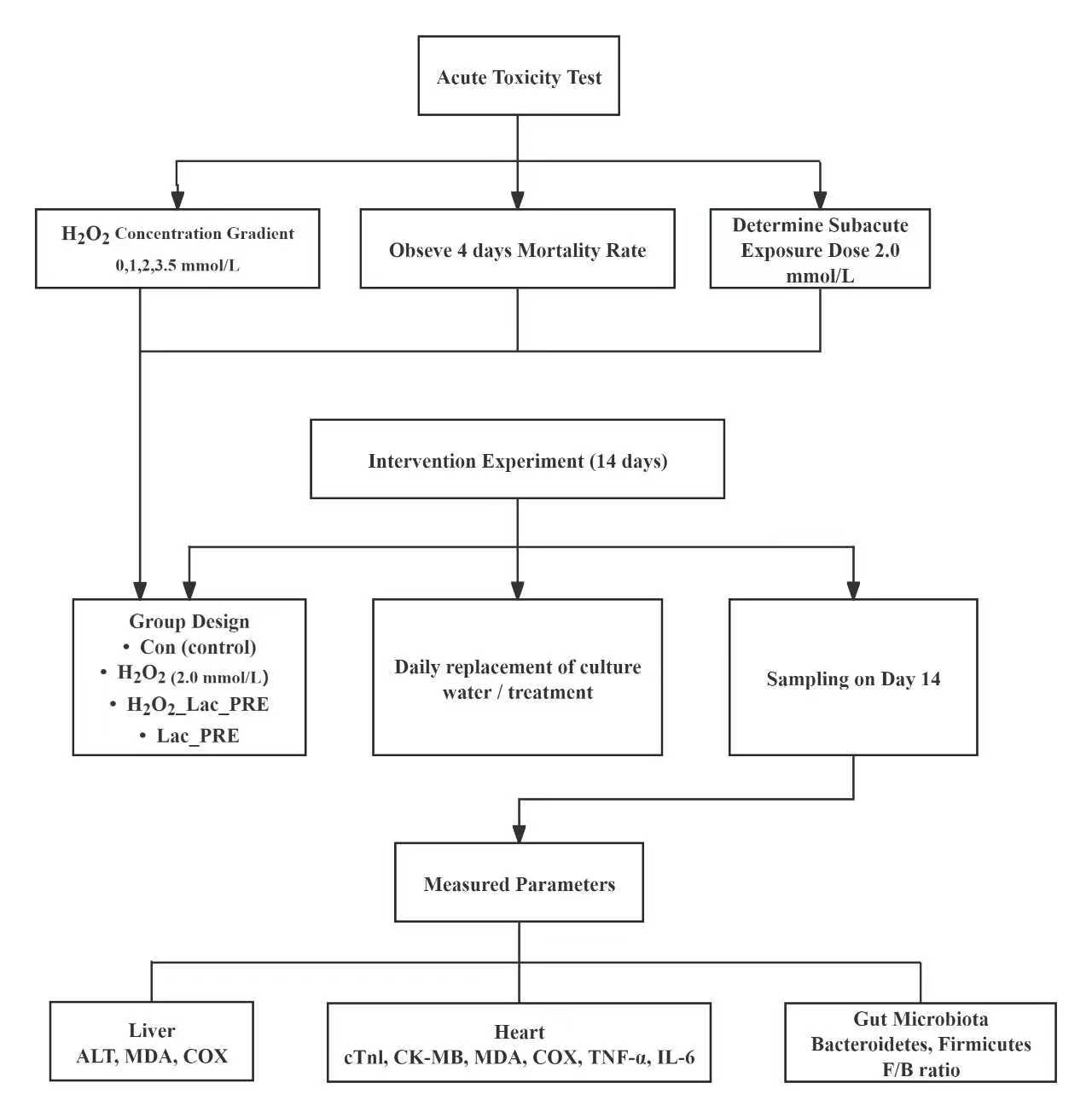

Supplement: Supplementary file 3 [file Image1.jpeg]

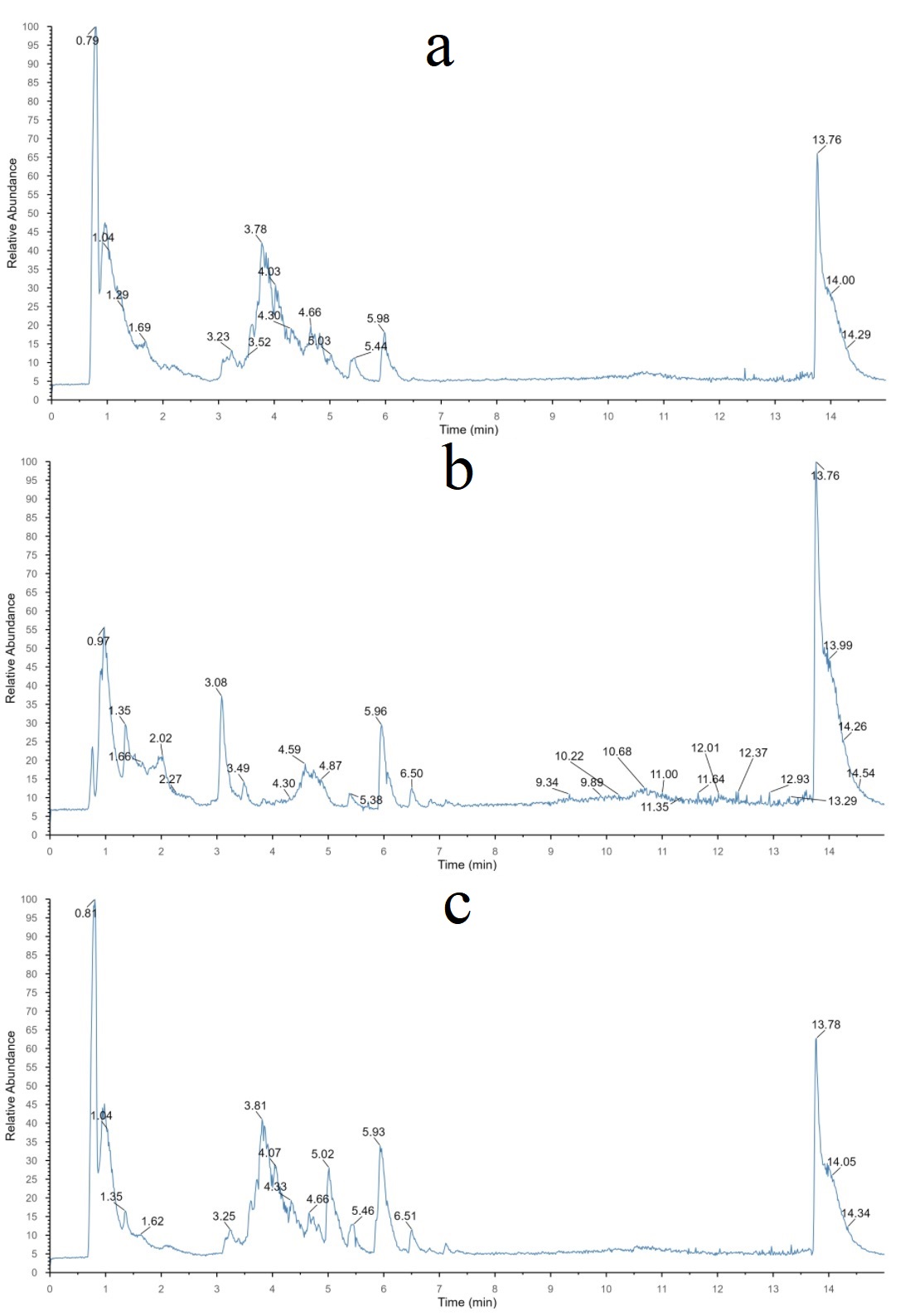

Supplement: Supplementary file 4 [file Image4.jpeg]

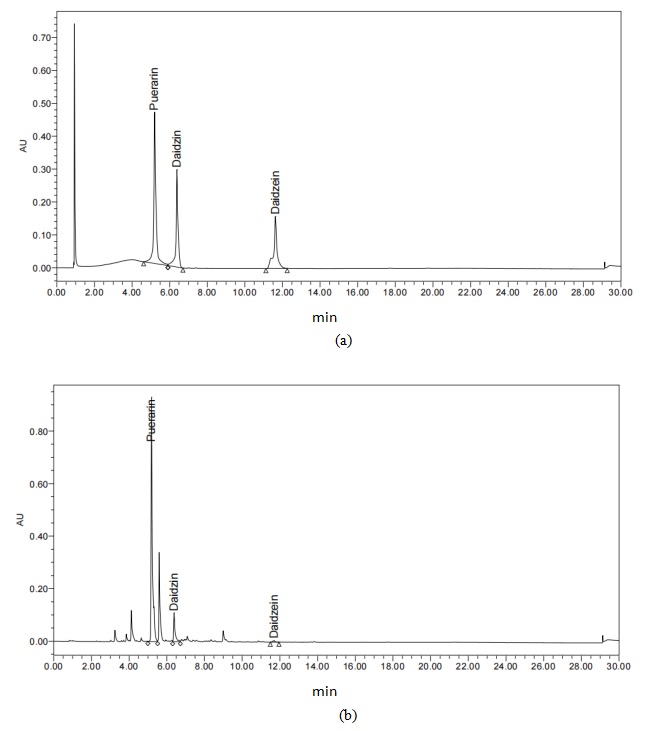

Supplement: Supplementary file 5 [file Image2.jpeg]

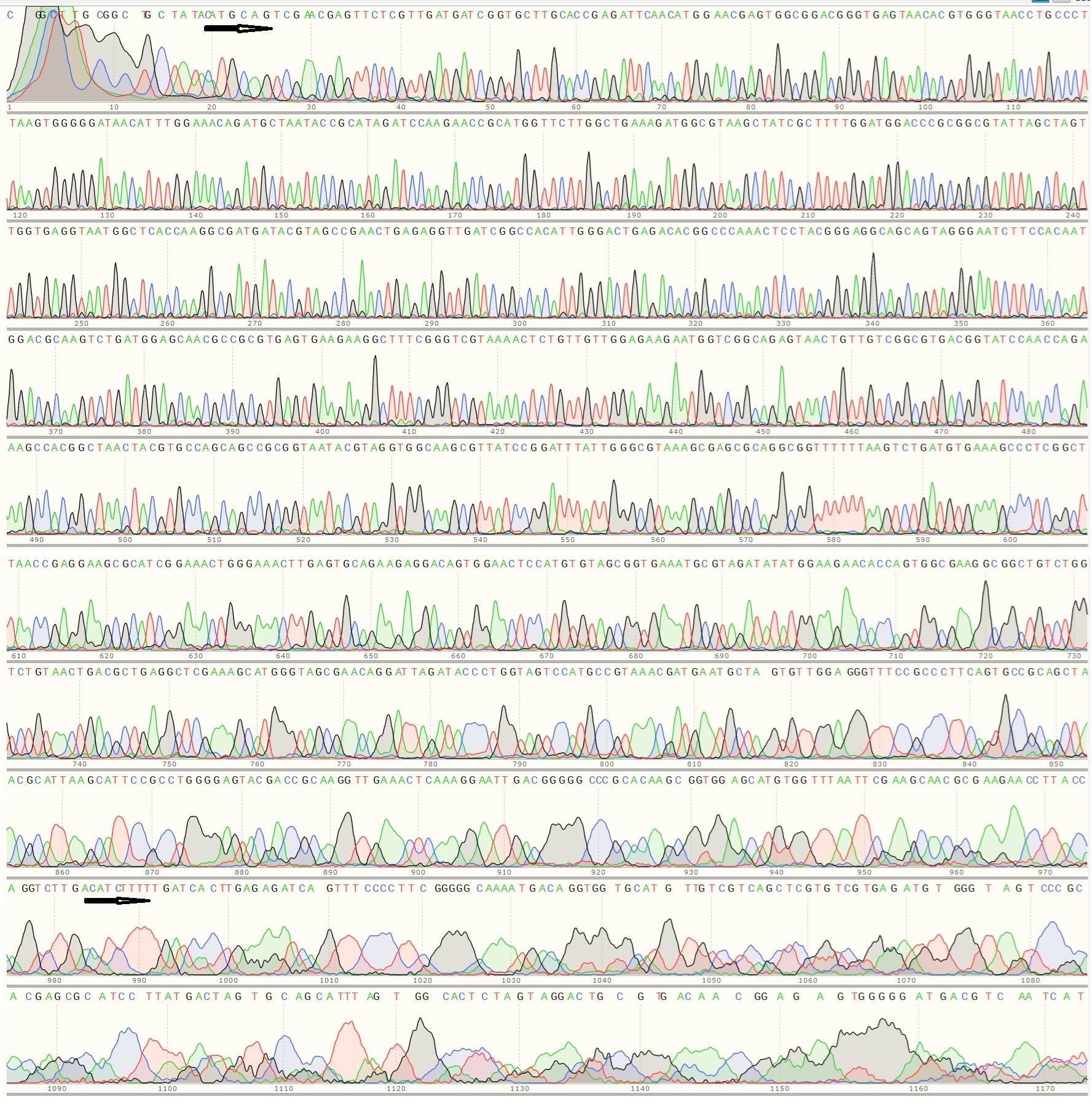

Supplement: Supplementary file 6 [file Image5.jpeg]
