# Supplementary material for: Ameliorative effects of a Lactobacillus paracasei and Puerariae Radix extract complex on hydrogen peroxide-induced oxidative damage in zebrafish
Source: Front Pharmacol. 2026 Jun 17;17:1787487. doi: 10.3389/fphar.2026.1787487 (PMC13318986; doi:10.3389/fphar.2026.1787487)
Supplement: Supplementary file 2 [file Table1.docx]

**Supplementary Table1.** Number of Detected Compounds in Each Group

| Group | Number of Compounds with Abundance > 0 | Proportion | Number of Samples |
| --- | --- | --- | --- |
| PRE | 1318 | 91.1% | 3 replicates |
| L. paracasei | 1298 | 89.8% | 3 replicates |
| Lac_PRE | 1350 | 98.2% | 3 replicates |
