# Supplementary material for: Ameliorative effects of a Lactobacillus paracasei and Puerariae Radix extract complex on hydrogen peroxide-induced oxidative damage in zebrafish
Source: Front Pharmacol. 2026 Jun 17;17:1787487. doi: 10.3389/fphar.2026.1787487 (PMC13318986; doi:10.3389/fphar.2026.1787487)
Supplement: Supplementary file 7 [file Table2.docx]

**Supplementary Table 2**. Unique Compounds Identified in the Lac_PRE Group

| Number | Description | Category | Mean Abundance |
| --- | --- | --- | --- |
| 1 | 5-Hydroxy-7,3',4'-trimethoxy-8-methylisoflavone 5-neohesperidoside | Flavonoids/Polyphenols | 9.29×10^5^ |
| 2 | Butyrolactone derivative | Other） | 5.10×10^5^ |
| 3 | Moracin H | Others | 7.96×10^4^ |
