# Supplementary material for: Ameliorative effects of a Lactobacillus paracasei and Puerariae Radix extract complex on hydrogen peroxide-induced oxidative damage in zebrafish
Source: Front Pharmacol. 2026 Jun 17;17:1787487. doi: 10.3389/fphar.2026.1787487 (PMC13318986; doi:10.3389/fphar.2026.1787487)
Supplement: Supplementary file 8 [file Table3.docx]

**Supplementary Table3.** Comparative analysis of 20 high-abundance metabolites

| Description | PRE | L. paracasei | Lac_PRE | H | P (Kruskal-Wallis) | Category |
| --- | --- | --- | --- | --- | --- | --- |
| Puerarin | (1.43 ± 0.05)×10⁸ | (2.32 ± 0.04)×10⁵ | (1.83 ± 0.08)×10⁸ | 7.20 | 0.027 | Flavonoids/Polyphenols |
| Daidzin | (1.63 ± 0.05)×10⁸ | (5.38 ± 3.42)×10⁴ | (1.97 ± 0.05)×10⁸ | 7.20 | 0.027 | Flavonoids/Polyphenols |
| 6''-O-Malonyldaidzin | (9.83 ± 0.63)×10⁷ | (1.58 ± 1.38)×10³ | (1.07 ± 0.03)×10⁸ | 7.32 | 0.026 | Flavonoids/Polyphenols |
| L-Proline | (3.39 ± 0.19)×10⁷ | (6.78 ± 0.22)×10⁶ | (3.34 ± 0.02)×10⁷ | 7.20 | 0.027 | Amino acids/Peptides |
| 3,3-Dimethylglutaric acid | (2.41 ± 0.06)×10⁸ | (4.57 ± 0.75)×10⁵ | (1.82 ± 0.10)×10⁸ | 7.20 | 0.027 | Others |
| Vitexin | (1.18 ± 0.04)×10⁸ | (4.66 ± 2.57)×10³ | (1.30 ± 0.06)×10⁸ | 7.32 | 0.026 | Others |
| Lauryldiethanolamine | (2.93 ± 0.37)×10⁷ | (5.14 ± 0.23)×10⁷ | (1.15 ± 0.06)×10⁸ | 7.20 | 0.027 | Others |
| 1-O-Sinapoylglucose | (7.77 ± 0.15)×10⁷ | (1.69 ± 0.25)×10⁵ | (1.07 ± 0.05)×10⁸ | 7.20 | 0.027 | Others |
| 6-Methoxymellein | (8.82 ± 0.26)×10⁷ | (5.84 ± 0.52)×10⁵ | (9.22 ± 0.43)×10⁷ | 7.32 | 0.026 | Others |
| Mirificin | (6.55 ± 0.17)×10⁷ | (4.00 ± 0.32)×10⁴ | (7.70 ± 0.36)×10⁷ | 7.20 | 0.027 | Others |
| Prunitrin | (5.43 ± 0.16)×10⁷ | (5.53 ± 0.35)×10⁴ | (6.48 ± 0.30)×10⁷ | 7.20 | 0.027 | Others |
| Glycitin | (4.46 ± 0.34)×10⁷ | (4.24 ± 3.66)×10³ | (4.86 ± 0.23)×10⁷ | 7.32 | 0.026 | Others |
| Glutathionyl-3-hydroxykynurenine glucoside | (4.69 ± 0.01)×10⁷ | 0 | (5.14 ± 0.13)×10⁷ | 8.00 | 0.018 | Others |
| Chrysophanol 8-gentiobioside | (4.44 ± 0.11)×10⁷ | (1.10 ± 0.82)×10³ | (4.80 ± 0.15)×10⁷ | 7.32 | 0.026 | Others |
| 1-Salicylate glucuronide | (4.34 ± 0.10)×10⁷ | (2.12 ± 0.14)×10⁵ | (4.50 ± 0.19)×10⁷ | 7.32 | 0.026 | Others |
| Formononetin | (5.00 ± 0.28)×10⁵ | (3.63 ± 0.20)×10⁵ | (3.76 ± 0.03)×10⁷ | 7.20 | 0.027 | Others |
| Enterolactone 3'-glucuronide | (3.13 ± 0.10)×10⁷ | 0 | (3.78 ± 0.09)×10⁷ | 8.00 | 0.018 | Others |
| Molludistin 2''-rhamnoside | (3.11 ± 0.20)×10⁷ | 0 | (3.31 ± 0.11)×10⁷ | 8.00 | 0.018 | Others |
| Rhamnazin 3-rutinoside | (2.91 ± 0.03)×10⁷ | (1.78 ± 3.08)×10² | (3.38 ± 0.12)×10⁷ | 8.00 | 0.018 | Others |
| Sphinganine | (1.66 ± 0.55)×10⁶ | (1.27 ± 0.01)×10⁷ | (3.23 ± 0.11)×10⁷ | 7.20 | 0.027 | Others |
