# Supplementary material for: Ameliorative effects of a Lactobacillus paracasei and Puerariae Radix extract complex on hydrogen peroxide-induced oxidative damage in zebrafish
Source: Front Pharmacol. 2026 Jun 17;17:1787487. doi: 10.3389/fphar.2026.1787487 (PMC13318986; doi:10.3389/fphar.2026.1787487)
Supplement: Supplementary file 9 [file Table4.docx]

**Supplementary Table 4.** Major chemical profiles and multi-component quantification of Puerariae Radix extract (PRE) by HPLC

| Component | Retention time (min) | Content (mg/g) | Number of Samples |
| --- | --- | --- | --- |
| Puerarin | 5.208 ± 0.010 | 2.468 ± 0.119 | 3 replicates |
| Daidzin | 6.389 ± 0.004 | 0.544 ± 0.020 | 3 replicates |
| Daidzein | 11.727 ± 0.009 | 0.153 ± 0.007 | 3 replicates |

Data are expressed as mean of three independent injections (RSD < 2.0%).
